# Invasive stink bug favors naïve plants: Testing the role of plant geographic origin in diverse, managed environments

**DOI:** 10.1038/srep32646

**Published:** 2016-09-01

**Authors:** Holly M. Martinson, Erik J. Bergmann, P. Dilip Venugopal, Christopher B. Riley, Paula M. Shrewsbury, Michael J. Raupp

**Affiliations:** 1 Department of Entomology, University of Maryland, College Park, MD 20742, USA; 2 AAAS S & T Policy Fellow, U.S. Environmental Protection Agency, Office of Air and Radiation, Office of Transportation & Air Quality, Washington DC 20460, USA; 3 Department of Entomology, The Ohio State University, Columbus, OH 43210, USA

## Abstract

With the introduction and establishment of exotic species, most ecosystems now contain both native and exotic plants and herbivores. Recent research identifies several factors that govern how specialist herbivores switch host plants upon introduction. Predicting the feeding ecology and impacts of introduced generalist species, however, remains difficult. Here, we examine how plant geographic origin, an indicator of shared co-evolutionary history, influences patterns of host use by a generalist, invasive herbivore, while accounting for variation in plant availability. The brown marmorated stink bug, *Halyomorpha halys*, is a highly polyphagous Asian herbivore and an economically important invasive pest in North America and Europe. In visual surveys of 220 plant taxa in commercial nurseries in Maryland, USA, *H. halys* was more abundant on non-Asian plants and selected these over Asian plants. The relationship between the relative use of plants and their availability was strongly positive but depended also on plant origin at two of our three sites, where the higher relative use of non-Asian plants was greatest for highly abundant taxa. These results highlight the importance of considering both plant origin and relative abundance in understanding the selection of host plants by invasive generalist herbivores in diverse, natural and urban forests.

With increased global trade, exotic species have become widespread and continue to pose serious threats to biodiversity worldwide^1^,^2^. Herbivorous insects are among the most economically and ecologically damaging exotic species, leading to billions of $US in losses and management costs annually^3^,^4^. As exotic plants also make up a substantial proportion of the species in most terrestrial ecosystems^5^, understanding and predicting how exotic herbivores will interact with native and exotic plant species in their invaded ranges is becoming increasingly important.

Several ecological and evolutionary arguments explicate how native and exotic plants and herbivores are likely to interact based on their geographic origins^6^,^7^,^8^. Most herbivorous insects are specialists, feeding on plants within a single family^9^. Because these herbivores may not have adaptations to feed on novel plants, host shifts may be limited^10^. Thus, exotic herbivores encountering a suite of potential host plants may select plants from their original range or close relatives of those plants in the newly invaded range.

On the other hand, plants with little or no shared co-evolutionary history with an herbivore may be more susceptible to herbivory from a novel consumer. The concept of evolutionary naiveté has received significant attention in the invasion literature and is integral to the formulation of hypotheses such as “biotic resistance”^11^, “new associations”^12^, and “defense free space”^8^. These hypotheses predict that an exotic consumer will preferentially use naïve resources in their invaded range, due to a lack of evolved defenses.

Recent work has demonstrated that the geographic origins of plants and herbivores are frequently important in determining resource consumption and ecological impacts in the invaded range, but consumer diet breadth and phylogenetic relationships among plants modify these expectations^13^,^14^. Specifically, monophagous and oligophagous consumers generally suffer fitness losses on novel host plants, especially when novel hosts are phylogenetically distant from their ancestral hosts^14^. Polyphagous consumers, on the other hand, often experience fitness gains on novel plants, regardless of phylogenetic relatedness among ancestral and novel hosts^14^.

Exotic generalist herbivores establish successfully, enjoy fitness gains in the invaded range, and have broad ecological impacts on diverse host plant taxa^15^,^16^. Understanding and predicting the use of host plants by exotic generalist herbivores tackles a formidable ecological and management problem. As a null hypothesis, a polyphagous consumer may feed according to the availability of resources in the landscape^17^. Deviations from this null expectation can indicate electivity, or selection for or against certain plant taxa given the available resources^18^. How resource availability and origin influence host plant use by introduced generalist consumers remains untested. Such knowledge is essential for predicting and mitigating the impacts of broadly feeding, introduced consumers.

Here, we test whether plant origin and availability singly or interactively influence the abundance and electivity of the herbivorous stink bug, *Halyomorpha halys* (Stål) (Hemiptera: Pentatomidae), across diverse trees and shrubs in commercial plant nurseries. Originally native to eastern Asia, *H. halys* is highly invasive and broadly polyphagous, feeding on and damaging field and vegetable crops, tree fruits, and ornamental and wild plants^19^,^20^. Using three years of repeated visual surveys of *H. halys*, we demonstrate that plant origin, while important, needs to be considered along with plant relative abundance to understand how invasive generalist consumers use novel resources.

## Results

### Effect of plant origin

**Figure 1:**
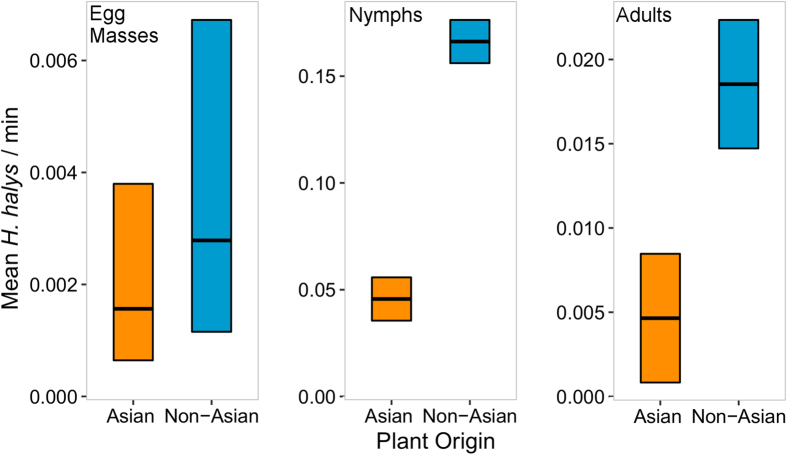
Mean abundances (number per 1 min. survey) of egg masses, nymphs, and adults of *Halyomorpha halys* for plants of non-Asian (blue) and Asian (orange) origins. The mean estimates were derived from Poisson-lognormal GLMM (egg masses) and LMEs (nymphs and adults). Plotted are back transformed model estimated means (thickened horizontal line) and SE (log link function for egg mass, and cube root transformation for nymphs and adults). Tukey’s post hoc comparisons are all significant at α = 0.05.

The abundance of each life stage of *H. halys* was significantly influenced by plant origin (Fig. 1). Mean abundance per survey was significantly higher on plant taxa of non-Asian compared with those of Asian origin for egg masses (Wald’s χ^2^ = 8.95, *df* = 1, *P* = 0.0028), nymphs (*F* test; values in parentheses denote total observations: *F*_(594)_ = 26.87, *P* < 0.0001), and adults (*F*_(594)_ = 10.91, *P* = 0.0010; Fig. 1).

### Relative use of plants

**Figure 2:**
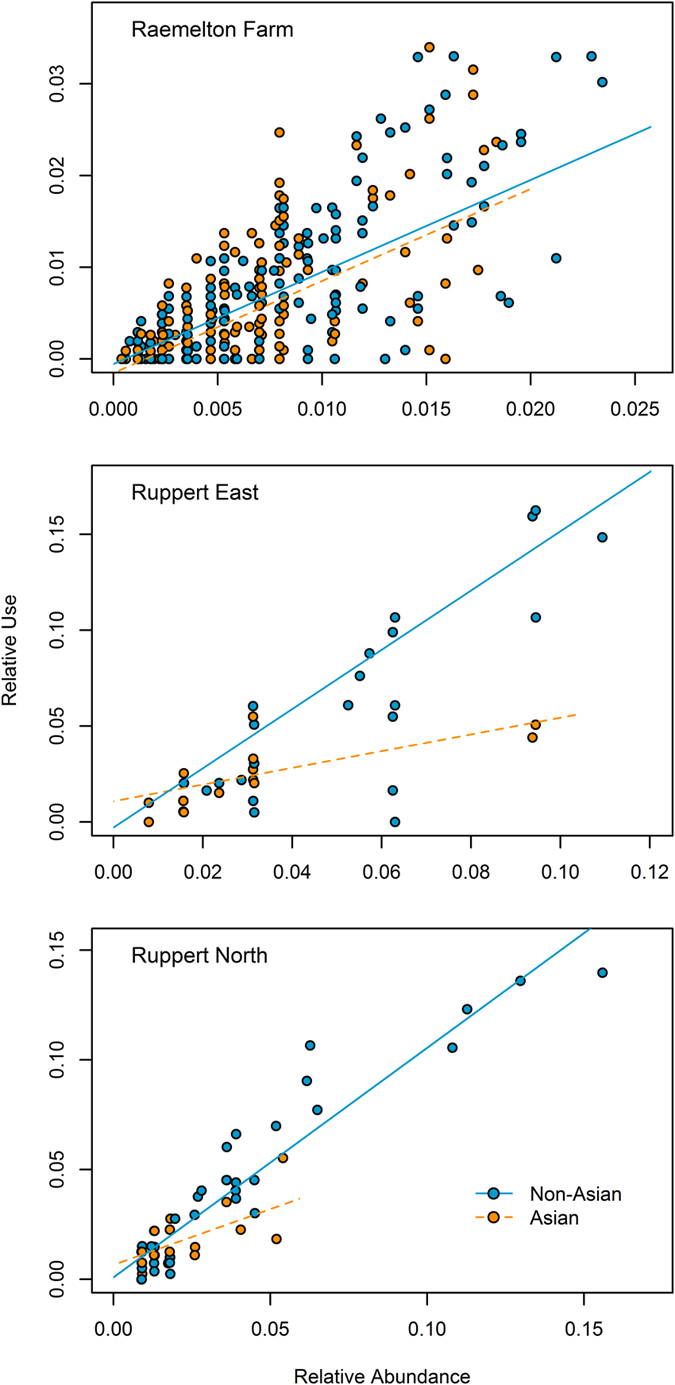
Relationships between relative abundance, relative use, and plant origin by site. Lines show relationships estimated from mixed models (incorporating random effects of year and taxonomic factors; see Methods for exact model specifications), and points show relative use for each plant taxon each year of sampling for non-Asian (blue symbols, solid lines) and Asian (orange symbols, dashed lines) taxa.

The relative use of plant taxa by *H. halys* at our study sites depended on the combination of plant relative abundance and origin (Fig. 2). At Raemelton Farm, our most diverse site, the relative use of plants was an increasing function of plant relative abundance and was higher for non-Asian compared to Asian plants (Fig. 2). The most parsimonious model included the additive fixed effects of plant origin (*F*_(496)_ = 11.09, *P* = 0.0009) and plant relative abundance (*F*_(496)_ = 537.97, *P* < 0.0001). At Ruppert East, the relationship between relative use and relative abundance depended on plant origin (significant interactive effect: *F*_(40)_ = 9.87, *P* = 0.0007; Fig. 2). Specifically, the slope of the relationship between relative use and relative abundance was steeper for plants with non-Asian (*b*_*1*_ = 1.43) compared to Asian (*b*_*1*_ = 0.55) origins. Similarly, a significant interaction between relative abundance and plant origin was evident at Ruppert North, such that the higher relative use of non-Asian plants became more pronounced with increasing plant relative abundance (significant interactive effect: *F*_(58)_ = 6.468, *P* = 0.0145; Fig. 2). Again, the slope of the relationship between relative use and relative abundance for non-Asian plants (*b*_*1*_ = 1.05) was steeper than that for Asian plants (*b*_*1*_ = 0.51). Because the ranges of *p* differed for Asian and non-Asian plants at this site, we examined a reduced data set of taxa for which *p* < 0.06 (the maximum for Asian plants). The interactive effect remained significant for this subset of the data, with an increasing difference in relative use between Asian and non-Asian taxa with increasing relative abundance (*F*_(51)_ = 10.75, *P* = 0.0022).

### Electivity

**Figure 3:**
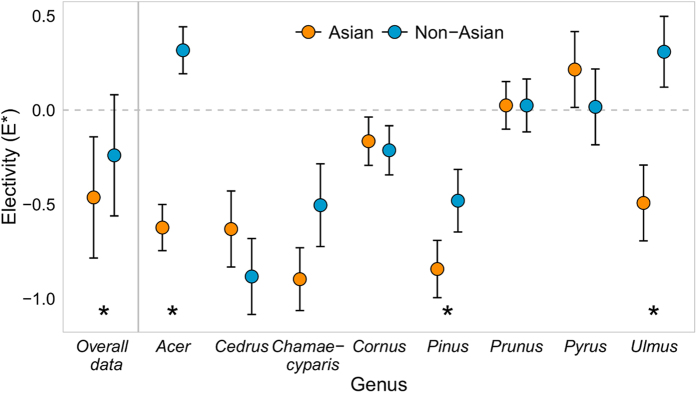
Electivity (E*) across the full data set (*Overall data*) and for the subset of genera with at least 2 taxa with Asian and 2 taxa with non-Asian origins (genus-level analysis). Plotted are model estimated means and SE; significance of Tukey’s post hoc comparisons within genera (*P* < 0.05) are indicated with an *.

Electivity was higher among taxa with non-Asian compared to Asian origins for the full data set (*F*_(594)_ = 31.48, *P* < 0.0001; Fig. 3, *Overall data*). For the genus-level analysis, the effect of plant origin on electivity depended strongly on plant genus (significant interactive effect: *F*_(212)_ = 9.77, *P* < 0.0001; Fig. 3). Tukey’s post hoc comparisons revealed significantly higher electivity for non-Asian than Asian taxa in the genera *Acer, Pinus*, and *Ulmus* (adjusted *P* < 0.05) and slightly higher electivity for non-Asian taxa in the genus *Chamaecyparis* (adjusted *P* = 0.097). In particular, non-Asian *Acer* and *Ulmus* were strongly selected by *H. halys* (positive values of E*) in these landscapes.

## Discussion

Accidental and purposeful introductions of both plants and herbivores have set the stage for a myriad of potential new consumer-resource interactions. Predicting interactions in such non-coevolved systems is a major challenge in modern ecology^21^. Our results identify the roles of both plant origin and abundance on use and electivity by the exotic generalist *H. halys*. Exotic consumers and pathogens are frequently able to exploit resources in their invaded ranges, often due to a lack of co-evolved defenses^8^. Prime examples of this phenomenon, however, have typically involved relatively specialized consumers: the introduced Eurasian viburnum leaf beetle, *Pyrrhalta viburni* (Paykull) exploits North American *Viburnum* spp L.^22^; the Asian emerald ash borer, *Agrilus planipennis* Fairmaire, is currently decimating North American ash trees (*Fraxinus* spp L.)^23^; and the introduced chestnut blight, *Cryphonectria parasitica* (Murrill) Barr, has eliminated American chestnut, *Castanea dentata* (Marshall) Borkh, from eastern forests^24^.

Whereas introduced specialist consumers may feed on resources which are phylogenetically or chemically similar to those found in their native ranges^13^,^14^, the diet of introduced generalist consumers is more difficult to predict. Due to their ability to tolerate a broad range of host defenses or suppress plant defenses, polyphagous herbivores may be even better able to make use of novel plants than specialists^25^,^26^. Thus, when polyphagous herbivores are introduced, they tend to incur fewer negative fitness consequences from feeding on naïve hosts^14^ and may generally prefer native plants^27^. In our three-year field study, the highly polyphagous *H. halys* was more abundant on plants not present in its native range (Fig. 1) and selected these over Asian plants (Fig. 2). Similarly, Morrison and Hay^28^ demonstrated that generalist apple snails and crayfish preferentially selected aquatic plants with which they shared little co-evolutionary history. Thus, in habitats with both exotic and native plants, it is clear that host shifting by introduced generalist herbivores is common and can be detrimental to the health of native plants. In the case of *H. halys*, which specializes in attacking reproductive organs of plants^29^, preference for plants native to the invaded realm portends broader ecological consequences such as invasional meltdown.

In addition to the geographic origin of plants, plant availability strongly influenced the relative use of plants by *H. halys* in this study and was a significant factor across all sites (Fig. 2). A null expectation for a generalist consumer encountering multiple potential resources would be a one-to-one relationship between relative use and relative abundance^17^, and this is the case for some polyphagous herbivores. Working with the polyphagous fall webworm, *Hyphantria cunea* (Drury), Mason *et al*.^30^ documented that host plant use in eastern forests was governed largely by the relative abundance of plants. Polyphagous species often, however, exhibit electivity, selecting or avoiding particular host plants and deviating from expectations based solely on plant availability^31^. In our study, variation around the expected one-to-one line (*i.e.*, electivity) was evident across all sites and in the data set as a whole, but the specific relationship between relative use and relative abundance differed among sites. Notably, the effects of plant origin and relative abundance were additive at our highly diverse site (Fig. 2, *Raemelton Farm*) but interactive at two less-diverse sites (Fig. 2, *Ruppert East, Ruppert North*; Table 1). Relative use of any given taxon by *H. halys* at the highly diverse Raemelton site was on average quite low (note the difference in axis scales among sites in Fig. 2), with extensive variation around the regression lines. We attribute such low values of relative use to the very high plant diversity at Raemelton, with each taxon contributing only a small portion of the total plant abundance at the site.

The importance of considering the joint effects of both plant abundance and plant origin is evident at the other two sites. There, plant relative use increased more steeply with plant abundance for non-Asian compared to Asian plants (Fig. 2), leading to large differences in use between abundant non-Asian and abundant Asian plants. We also found evidence that *H. halys* selects non-Asian taxa in the genera *Acer* L. and *Ulmus* L. (Fig. 3), trees which can be exploited for sugar rich exudates even when reproductive structures are not present^32^. Together, our results suggest that the risk posed by *H. halys* to trees may be exacerbated for abundant maples (*Acer* spp), elms (*Ulmus* spp), and pines (*Pinus* spp L.) in eastern forests (Fig. 3) particularly in low diversity environments such as the urban forest. This finding, which bears investigation at additional sites and in other habitats invaded by *H. halys*, may indicate increased risk for dominant native trees. Increasing the diversity of tree species and cultivars in managed environments such as urban forests and landscapes may be increasingly important in buffering against losses to introduced insect pests^33^. Furthermore, our results highlight the importance of jointly considering the geographic origin and relative abundance of potential resources when assessing novel consumer-resource interactions in invaded habitats.

## Methods

### Field surveys

We conducted repeated one-minute visual surveys of trees and shrubs in commercial nurseries in Maryland, USA, from late May through early August, 2011–2013. In each survey, we recorded the number of egg masses, nymphs, and adults of *H. halys* on the leaves, fruits and flowers, and bark of the tree up to a height of 3 m. In 2011, we conducted four surveys per tree at Raemelton Farm in Frederick County, MD (39.299468°N, 77.458549°W). In 2012 and 2013, we conducted six surveys per tree and included two additional sites at Ruppert Nurseries in Montgomery County, MD (Ruppert East: 39.238821°N, 77.138334°W; Ruppert North: 39.251091°N, 77.157074°W). At each site, trees of a particular species or cultivated variety (hereafter ‘taxa’) are planted within a row of 25–35 trees, but different rows within a field are typically planted with different taxa. For 3–4 fields per site each year, we conducted spatially stratified surveys on three edge trees (0–5 m from the field edge) and three interior (15–20 m from the edge) trees in each row to mitigate the influence of known edge effects^34^,^35^,^36^.

### Definitions and data set construction

We calculated the abundance, relative use, and electivity of *H. halys* separately for each plant taxon. Because the available plants varied across sites and over time as trees were sold and replanted, all calculations were made for taxa from each site and year separately. Thus, the experimental unit throughout the analysis is a unique plant taxon at a site in a year. To calculate the abundance of *H. halys* for each taxon, we summed the abundance of each life stage (egg masses, nymphs, and adults separately) across all surveys conducted on each plant taxon within a year for each site. These summed abundances were used used in conjunction with the total number of surveys conducted for each taxon to calulate the mean number of individuals (or egg masses) per 1-min. survey.

We calculated the relative abundance (*p*) of each plant taxon as the total number of individuals of the taxon divided by the total number of individual trees of all taxa surveyed at a site for a given year. This measure of plant availability assumes that the number of trees surveyed is an unbiased sample of all the trees at the site, a reasonable assumption given that we equally surveyed all rows of a field, and that the sampled fields were representative of the plant diversity and composition at each site. We calculated the relative use (*r*) of each taxon as the number of trees of the taxon that were used by *H. halys*, divided by the total number of trees used by *H. halys* at a site in a year. Use of a taxon was defined as the presence of any life stage on a tree of that taxon during any of the sampling periods within a year. As with abundance, both *p* and *r* were calculated within each site each year.

Electivity measures the relative use of resources after accounting for their relative abundances^17^,^18^. We calculated indices of electivity for each plant taxon *i* at each site in each year using *r* and *p*. We used Vanderploeg and Scavia’s relativized electivity index, 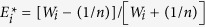, where 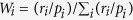 and *n* is the number of plant taxa available^37^. E* was the most suitable electivity index for our purposes because: 1) it best met model assumptions for our analysis; 2) it is less sensitive than the forage ratio, *FR*_*i*_ = *r*_*i*_/*p*_*i*_, and Ivelev’s electivity, *E*_*i*_ =(*r*_*i*_ − *p*_*i*_)/(*r*_*i*_ + *p*_*i*_)^18^, to values of *n*; and 3) it has a straight-forward interpretation, ranging in value from negative one for resources which are completely avoided, to positive one for resources which are used exclusively. All values of E*, *E*, and *FR* were highly correlated in our data set (Pearson’s *ρ* > 0.93, *P* < 0.0001 for pairwise correlations; see also Lechowicz^37^).

Plant origin (following Dirr^38^) was assigned as follows: taxa originally from the native range of *H. halys (i.e.*, China, Taiwan, Japan, Korea^19^) were considered “Asian”; taxa not originally from that range were considered “non-Asian.” Taxa with hybrid Asian and non-Asian origins or with unknown origins (*i.e.*, open-pollinated *Malus* taxa) were used in calculations of *r, p*, and E* but were dropped from the analysis of plant origin. In total, we conducted 47,136 one-minute surveys on 4,620 unique trees. Excluding species of hybrid or unknown origins yielded a final data set of 594 observations of the mean abundance, relative use, and electivity of *H. halys* on 220 unique taxa from 58 genera of plants. Please see Table 1 for a summary of the sampling effort at each site and Supplementary Table S1 for the full data table.

### Statistical analysis

All statistical analyses were conducted in the program R^39^. To assess the effects of plant origin on the abundance of *H. halys*, we constructed separate mixed effects models for egg masses, nymphs, and adults using the *lme4* package^40^. Model building and selection procedures for the mixed effects modeling followed the approach used by Zuur *et al*.^41^. For each model, we considered a set of random effects that included the following terms: plant classification (angiosperm or gymnosperm), plant genus, genus within classification category, plant taxon, and site-year combination. We then selected the best set of random effects as the model with the lowest AIC (Akaike’s Information Criterion) value prior to the assessment of fixed effects.

For the analysis of egg mass abundance, we constructed a generalized linear mixed model (GLMM) based on Laplace approximation, with a Poisson-lognormal error distribution and log link function^42^,^43^. This GLMM included the following terms: total egg mass abundance per plant taxon at a site in a year as the response variable, an offset for the number of surveys conducted for each plant taxon, and the fixed effect of plant origin. The final set of random effects for this model included the site-year combination to account for repeated measures and an observation-level variable to account for overdispersion (see supplementary material in Bolker *et al*.^43^). We found no evidence for additional oversdispersion or correlations among random effect terms in the final model, and visual plots of the variances in a location-scale plot with superimposed loess fit demonstrated that the assumptions of the model were met appropriately. The statistical significance of this egg mass GLMM model was determined with a Wald’s chi-square test; significance for all tests was evaluated using an α-level threshold of 0.05.

For the abundance of nymphs and adults, we constructed separate linear mixed effects models (LME’s). The response variable of mean abundance per one-minute survey was cube-root transformed to normalize residuals for the nymph and adult abundance analyses. The appropriateness of the final models was confirmed using diagnostic plots visualizing within-group residuals (standardized residuals vs. fitted values, normal Q-Q plots, and histograms of residuals) and estimated random effects (normal Q-Q plots and pairs-scatterplot matrix)^40^. Final random effects for both nymph and adult models included genus within classification and site-year combination. We used *F* tests to assess the significance of fixed effects and compared model estimated means through Tukey’s post hoc tests.

We next modeled relative use as a function of the fixed effects of plant relative abundance, plant origin, and their interaction. Sites were analyzed separately due to widely differing plant diversity and ranges of relative abundance and relative use (see Table 1); for Raemelton Farm, the best model fit was achieved by square root transforming values for relative use and relative abundance. For each site, we again selected the most parsimonious set of random effects via comparison of AIC values and then evaluated the significance of fixed effects with *F* tests. The best models for both Raemelton Farm and Ruppert East included random effects terms for genus within classification and year; the model for Ruppert North included the random effect of plant taxon.

For electivity, we first performed a global analysis of the full data set, comparing E* of Asian and non-Asian plants. Random effects for the final model included site-year combination and genus within classification. Because previous investigations found substantial differences in the mean abundance of *H. halys* among cultivars and species^36^,^44^, we next performed a genus-level analysis, asking whether differences in electivity between Asian and non-Asian plants were consistent across genera. For this analysis, we used the subset of genera with at least two taxa of Asian origin and two of non-Asian origin; all such genera were represented only at Raemelton Farm. We rescaled *r* and *p* to sum to 1 for each year for this subset of taxa only, and recalculated E*. The values of E* for this subset were highly correlated with those from the full data set (Pearson’s *ρ* = 0.9995, *P* < 0.0001) but allow a clearer test of selection for and against these particular taxa. Final random effects for this model included terms for year and plant classification.

## Additional Information

**How to cite this article**: Martinson, H. M. *et al*. Invasive stink bug favors naïve plants: Testing the role of plant geographic origin in diverse, managed environments. *Sci. Rep.* **6**, 32646; doi: 10.1038/srep32646 (2016).

## Supplementary Material

Supplementary Information

## Figures and Tables

**Table 1:** Summary of sampling across plant genera and taxa (species and cultivated varieties) and the mean (min, max) relative abundance (*p*), relative use (*r*), and electivity (E*) of plants in Maryland nurseries by *Halyomorpha halys* for each site and year.

Site	Year	Genera	Taxa	*p*	*r*	E*
Raemelton Farm	2011	54	174	0.006 (0.001, 0.02)	0.006 (0, 0.025)	−0.104 (−1, 0.241)
	2012	52	172	0.006 (0, 0.023)	0.006 (0, 0.043)	−0.283 (−1, 0.566)
	2013	55	196	0.005 (0, 0.023)	0.005 (0, 0.034)	−0.239 (−1, 0.469)
Ruppert East	2012	15	23	0.043 (0.016, 0.109)	0.043 (0.005, 0.159)	−0.081 (−0.835, 0.347)
	2013	17	25	0.04 (0.008, 0.094)	0.04 (0, 0.162)	−0.093 (−1, 0.293)
Ruppert North	2012	16	26	0.038 (0.013, 0.156)	0.038 (0.004, 0.14)	−0.054 (−0.557, 0.261)
	2013	20	39	0.026 (0.009, 0.113)	0.026 (0, 0.123)	−0.076 (−1, 0.27)

Relative use and electivity were calculated based on the presence of any life stage of *H. halys* on trees.
