# Supplementary material for: Invasive stink bug favors naïve plants: Testing the role of plant geographic origin in diverse, managed environments
Source: Sci Rep. 2016 Sep 1;6:32646. doi: 10.1038/srep32646 (PMC5007670; doi:10.1038/srep32646)
Supplement: Supplementary Information [file srep32646-s1.pdf]

Invasive stink bug favors naïve plants: Testing the role of plant geographic origin in diverse, managed environments

Holly M. Martinson[1], Erik J. Bergmann[1], P. Dilip Venugopal[1, 2, ¥], Christopher B. Riley[3], Paula M. Shrewsbury[1], and Michael J. Raupp[1]

[1] Department of Entomology, University of Maryland, College Park, MD 20742

[2] AAAS S&T Policy Fellow, U.S. Environmental Protection Agency, Office of Air and Radiation, Office of Transportation & Air Quality, Washington DC 20460

[3] Department of Entomology, The Ohio State University, Columbus, OH 43210

*Corresponding Author: hmartinson@alumni.duke.edu

[¥] The views presented here are solely those of the authors and do not represent the views of either AAAS or the U.S. Government.

	Site	Year	Plant Taxon	Classification	Origin	Egg masses per 1 min survey	Nymphs per 1 min survey	Adults per 1 min survey	*p*	*r*	*E**
1	Raemelton Farm	2011	Abies koreana	Gymnosperm	Asian	0	0	0.125	0.0071	0.0026	-0.4201
2	Raemelton Farm	2011	Acer campestre Evelyn	Angiosperm	Non Asian	0.0333	0.4833	1.7667	0.0089	0.0123	0.2078
3	Raemelton Farm	2011	Acer davidii	Angiosperm	Asian	0	0	0	0.0006	0	-1
4	Raemelton Farm	2011	Acer griseum	Angiosperm	Asian	0	0.8167	0.3	0.0089	0.0088	0.0426
5	Raemelton Farm	2011	Acer palmatum Bloodgood	Angiosperm	Asian	0.0417	0	1.0417	0.0036	0.0053	0.2405
6	Raemelton Farm	2011	Acer palmatum Moonfire	Angiosperm	Asian	0	0	0	0.0018	0	-1
7	Raemelton Farm	2011	Acer palmatum Red Emperor	Angiosperm	Asian	0.0455	0	0.2955	0.0065	0.0035	-0.2547
8	Raemelton Farm	2011	Acer palmatum Sango Kaku	Angiosperm	Asian	0	0	0	0.0018	0	-1
9	Raemelton Farm	2011	Acer palmatum var. dissectum Crimson Queen	Angiosperm	Asian	0	0	0	0.0036	0	-1
10	Raemelton Farm	2011	Acer pensylvanicum	Angiosperm	Non Asian	0.0313	2	0.5313	0.0047	0.0053	0.1012
11	Raemelton Farm	2011	Acer rubrum Bowhall	Angiosperm	Non Asian	0.0556	5.6944	0.4167	0.0053	0.0079	0.2405
12	Raemelton Farm	2011	Acer rubrum Brandywine	Angiosperm	Non Asian	0.0606	2.9545	0.553	0.0195	0.0245	0.1618
13	Raemelton Farm	2011	Acer rubrum Franksred	Angiosperm	Non Asian	0.1061	2.7879	0.6894	0.0195	0.0237	0.144
14	Raemelton Farm	2011	Acer rubrum October Glory	Angiosperm	Non Asian	0	5.9167	0.9722	0.0053	0.0079	0.2405
15	Raemelton Farm	2011	Acer rufinerve	Angiosperm	Asian	0	0	0	0.0018	0	-1
16	Raemelton Farm	2011	Acer saccharum Commemoration	Angiosperm	Non Asian	0	2	0.0833	0.0018	0.0026	0.2405
17	Raemelton Farm	2011	Acer saccharum Green Mountain	Angiosperm	Non Asian	0.0172	2.2759	0.3448	0.0172	0.0193	0.1068
18	Raemelton Farm	2011	Acer saccharum Legacy	Angiosperm	Non Asian	0.05	2.475	0.05	0.0059	0.007	0.133
19	Raemelton Farm	2011	Acer truncatum	Angiosperm	Asian	0	0	0.1111	0.0053	0.0018	-0.4674
20	Raemelton Farm	2011	Acer x freemanii Jeffersred	Angiosperm	Non Asian	0	6.375	0.6667	0.0036	0.0053	0.2405
21	Raemelton Farm	2011	Acer x tegmentosum White Tigress	Angiosperm	Asian	0	1.8333	0.0278	0.0053	0.0053	0.0426
22	Raemelton Farm	2011	Aesculus x carnea Briotii	Angiosperm	Non Asian	0	0.0625	0.3125	0.0071	0.0061	-0.0241
23	Raemelton Farm	2011	Aesculus x carnea Fort McNair	Angiosperm	Non Asian	0	0.375	0.9583	0.0071	0.0079	0.1012
24	Raemelton Farm	2011	Amelanchier x grandiflora Autumn Brilliance	Angiosperm	Non Asian	0	1.1818	0.4318	0.0065	0.0079	0.144
25	Raemelton Farm	2011	Amelanchier x grandiflora Princess Diana	Angiosperm	Non Asian	0	0.5417	0.1458	0.0071	0.0096	0.1991
26	Raemelton Farm	2011	Betula nigra BNMTF	Angiosperm	Non Asian	0	0.75	0.5556	0.0053	0.0053	0.0426
27	Raemelton Farm	2011	Betula papyrifera Renci	Angiosperm	Non Asian	0.0278	0.8333	0.8056	0.0053	0.0053	0.0426
28	Raemelton Farm	2011	Carpinus betulus Fastigiata	Angiosperm	Non Asian	0.0259	0.5	0.1983	0.0172	0.0149	-0.0217
29	Raemelton Farm	2011	Carpinus betulus Frans Fontaine	Angiosperm	Non Asian	0	0.1042	0.4792	0.0071	0.0061	-0.0241
30	Raemelton Farm	2011	Carya illinoinensis Choctaw	Angiosperm	Non Asian	0	0.5833	4.6667	0.0018	0.0026	0.2405
31	Raemelton Farm	2011	Cedrus atlantica Glauca	Gymnosperm	Non Asian	0	0	0.0833	0.0036	0.0018	-0.2949
32	Raemelton Farm	2011	Cedrus atlantica Kroh's Twisted	Gymnosperm	Non Asian	0	0	0	0.0018	0	-1
33	Raemelton Farm	2011	Cedrus deodara Shalimar	Gymnosperm	Asian	0	0	0.25	0.0036	0.0026	-0.1009
34	Raemelton Farm	2011	Celtis koraiensis	Angiosperm	Asian	0	0.0833	0.5	0.0018	0.0026	0.2405
35	Raemelton Farm	2011	Cercidiphyllum japonicum	Angiosperm	Asian	0.0167	2.75	0.7167	0.0089	0.0088	0.0426
36	Raemelton Farm	2011	Cercis canadensis	Angiosperm	Non Asian	0.0417	1.5417	0.4375	0.0071	0.007	0.0426
37	Raemelton Farm	2011	Cercis canadensis Alba	Angiosperm	Non Asian	0	1.7917	4.5833	0.0036	0.0053	0.2405
38	Raemelton Farm	2011	Cercis canadensis Appalachian Red	Angiosperm	Non Asian	0.0833	0.875	0.4167	0.0036	0.0035	0.0426
39	Raemelton Farm	2011	Cercis canadensis Covey	Angiosperm	Non Asian	0	1.1111	0.0278	0.0053	0.0053	0.0426
40	Raemelton Farm	2011	Cercis canadensis Forest Pansy	Angiosperm	Non Asian	0.0167	1.1833	1.3333	0.0089	0.0088	0.0426
41	Raemelton Farm	2011	Cercis canadensis Pink Heartbreaker	Angiosperm	Non Asian	0	2	1.625	0.0036	0.0053	0.2405
42	Raemelton Farm	2011	Chamaecyparis nootkatensis Pendula	Gymnosperm	Non Asian	0	0	0.9583	0.0036	0.0035	0.0426

	Site	Year	Plant Taxon	Classification	Origin	Egg masses per 1 min survey	Nymphs per 1 min survey	Adults per 1 min survey	*p*	*r*	*E**
43	Raemelton Farm	2011	Chamaecyparis obtusa Crippsii	Gymnosperm	Asian	0	0	0	0.0018	0	-1
44	Raemelton Farm	2011	Chionanthus retusus	Angiosperm	Asian	0	0	0	0.0018	0	-1
45	Raemelton Farm	2011	Cladrastis kentukea	Angiosperm	Non Asian	0.0833	3.1481	0.7963	0.016	0.0219	0.204
46	Raemelton Farm	2011	Cladrastis kentukea Perkins Pink	Angiosperm	Non Asian	0.25	3.75	0.25	0.0036	0.0044	0.153
47	Raemelton Farm	2011	Cornus controversa	Angiosperm	Asian	0	0.0417	0.375	0.0036	0.0044	0.153
48	Raemelton Farm	2011	Cornus florida	Angiosperm	Non Asian	0	0.25	0.75	0.0018	0.0026	0.2405
49	Raemelton Farm	2011	Cornus florida Appalachian Spring	Angiosperm	Non Asian	0	0.0833	0	0.0036	0.0009	-0.572
50	Raemelton Farm	2011	Cornus florida Cherokee Princess	Angiosperm	Non Asian	0	0.525	0.3125	0.0118	0.0079	-0.1527
51	Raemelton Farm	2011	Cornus florida Cloud 9	Angiosperm	Non Asian	0	0.1667	0.0833	0.0036	0.0018	-0.2949
52	Raemelton Farm	2011	Cornus florida COMCO #1	Angiosperm	Non Asian	0	0.625	0.3056	0.0107	0.0096	-0.0009
53	Raemelton Farm	2011	Cornus florida Jean's Appalachian Snow	Angiosperm	Non Asian	0	0.0417	0.9583	0.0036	0.0026	-0.1009
54	Raemelton Farm	2011	Cornus florida Kay's Appalachian Mist	Angiosperm	Non Asian	0	0.0417	0.375	0.0036	0.0026	-0.1009
55	Raemelton Farm	2011	Cornus florida x kousa Celestial	Angiosperm	Hybrid	0	0	0.5	0.0018	0.0009	-0.2949
56	Raemelton Farm	2011	Cornus florida x kousa Constellation	Angiosperm	Hybrid	0	0.875	0.8125	0.0024	0.0026	0.1012
57	Raemelton Farm	2011	Cornus florida x kousa Stellar Pink	Angiosperm	Hybrid	0.0125	0.375	0.2625	0.0118	0.0105	-0.0101
58	Raemelton Farm	2011	Cornus kousa Madison	Angiosperm	Asian	0	0	0.25	0.003	0.0026	-0.0101
59	Raemelton Farm	2011	Cornus kousa National	Angiosperm	Asian	0	0.3611	0.1944	0.0053	0.0053	0.0426
60	Raemelton Farm	2011	Cornus kousa Radiant Rose	Angiosperm	Asian	0	0	0	0.0006	0	-1
61	Raemelton Farm	2011	Cornus kousa Santomi	Angiosperm	Asian	0	0.2188	0.125	0.0142	0.0061	-0.3546
62	Raemelton Farm	2011	Cornus kousa var chinensis Milky Way	Angiosperm	Asian	0	0.0417	0	0.0036	0.0009	-0.572
63	Raemelton Farm	2011	Cornus macrophylla	Angiosperm	Asian	0.125	1.7083	0.7917	0.0036	0.0053	0.2405
64	Raemelton Farm	2011	Cornus officinalis	Angiosperm	Asian	0	0.125	0.75	0.0036	0.0044	0.153
65	Raemelton Farm	2011	Cornus walteri	Angiosperm	Non Asian	0	0	1	0.0018	0.0018	0.0426
66	Raemelton Farm	2011	Crataegus crusgalli Cruzam	Angiosperm	Non Asian	0	0	0.0833	0.0018	0.0009	-0.2949
67	Raemelton Farm	2011	Crataegus laevigata Superba	Angiosperm	Non Asian	0	1.9167	0.4167	0.0018	0.0018	0.0426
68	Raemelton Farm	2011	Crataegus phaenopyrum	Angiosperm	Non Asian	0	0	0.0833	0.0018	0.0009	-0.2949
69	Raemelton Farm	2011	Crataegus viridis Winter King	Angiosperm	Non Asian	0	0.0833	0.25	0.0089	0.0061	-0.1349
70	Raemelton Farm	2011	Cryptomeria japonica Yoshino	Gymnosperm	Asian	0	0	1.6	0.0089	0.0114	0.1721
71	Raemelton Farm	2011	Ficus carica Chicago Hardy	Angiosperm	Non Asian	0	0.0833	5	0.0018	0.0026	0.2405
72	Raemelton Farm	2011	Ginkgo biloba Autumn Gold	Gymnosperm	Asian	0.0139	0	0.2361	0.0107	0.007	-0.1588
73	Raemelton Farm	2011	Ginkgo biloba Magyar	Gymnosperm	Asian	0	0	0.7083	0.0036	0.0035	0.0426
74	Raemelton Farm	2011	Ginkgo biloba Princeton Sentry	Gymnosperm	Asian	0.1389	1.3333	0.0833	0.0053	0.0044	-0.0485
75	Raemelton Farm	2011	Gleditsia triacanthos Shademaster	Angiosperm	Non Asian	0.025	1.1917	1.9417	0.0178	0.021	0.133
76	Raemelton Farm	2011	Halesia tetraptera	Angiosperm	Non Asian	0.0417	0.7222	0.5556	0.0107	0.0131	0.153
77	Raemelton Farm	2011	Halesia tetraptera Arnold Pink	Angiosperm	Non Asian	0	0.1667	0.4167	0.0018	0.0018	0.0426
78	Raemelton Farm	2011	Hamamelis x intermedia Arnold Promise	Angiosperm	Hybrid	0	0	0.375	0.0036	0.0035	0.0426
79	Raemelton Farm	2011	Hamamelis x intermedia Diane	Angiosperm	Hybrid	0	0	0.0833	0.0018	0.0009	-0.2949
80	Raemelton Farm	2011	Hamamelis x intermedia Jelena	Angiosperm	Hybrid	0	0	0	0.0018	0	-1
81	Raemelton Farm	2011	Hamamelis x intermedia Pallida	Angiosperm	Hybrid	0	0	0	0.0018	0	-1
82	Raemelton Farm	2011	Heptacodium miconioides	Angiosperm	Asian	0	0.1875	0.2813	0.0047	0.0044	0.0103
83	Raemelton Farm	2011	Hibiscus syriacus Blue Bird	Angiosperm	Asian	0	0.4167	3.9167	0.0018	0.0026	0.2405
84	Raemelton Farm	2011	Hibiscus syriacus Diana	Angiosperm	Asian	0	0.0833	0.6667	0.0018	0.0026	0.2405

	Site	Year	Plant Taxon	Classification	Origin	Egg masses per 1 min survey	Nymphs per 1 min survey	Adults per 1 min survey	*p*	*r*	*E**
85	Raemelton Farm	2011	Hibiscus syriacus Red Heart	Angiosperm	Asian	0	2.2083	2.875	0.0036	0.0053	0.2405
86	Raemelton Farm	2011	Hibiscus syriacus Satin Blue	Angiosperm	Asian	0	1	4.8333	0.0036	0.0053	0.2405
87	Raemelton Farm	2011	Hibiscus syriacus Satin Rose	Angiosperm	Asian	0	1	0.625	0.0012	0.0018	0.2405
88	Raemelton Farm	2011	Hibiscus syriacus White Chiffon	Angiosperm	Asian	0	0.25	0	0.0006	0.0009	0.2405
89	Raemelton Farm	2011	Ilex opaca Jersey Princess	Angiosperm	Non Asian	0	0.3333	0	0.0018	0.0018	0.0426
90	Raemelton Farm	2011	Koelreuteria paniculata	Angiosperm	Asian	0.0484	1.4839	1.5081	0.0184	0.0237	0.1745
91	Raemelton Farm	2011	Larix leptolepis	Gymnosperm	Asian	0	0	0.125	0.0012	0.0009	-0.1009
92	Raemelton Farm	2011	Liquidambar styraciflua	Angiosperm	Non Asian	0.125	2.6667	1.7917	0.0036	0.0053	0.2405
93	Raemelton Farm	2011	Liquidambar styraciflua Hapdell	Angiosperm	Non Asian	0	0	1.25	0.0018	0.0026	0.2405
94	Raemelton Farm	2011	Liquidambar styraciflua Moraine	Angiosperm	Non Asian	0.0417	0.5972	0.5694	0.0107	0.014	0.1843
95	Raemelton Farm	2011	Liquidambar styraciflua Rotundiloba	Angiosperm	Non Asian	0.0833	0	0	0.0018	0.0009	-0.2949
96	Raemelton Farm	2011	Magnolia liliiflora x stellata Ann	Angiosperm	Asian	0	0	0.4167	0.0018	0.0026	0.2405
97	Raemelton Farm	2011	Magnolia liliiflora x stellata Merrill	Angiosperm	Asian	0	0	0.1667	0.0018	0.0009	-0.2949
98	Raemelton Farm	2011	Magnolia x loebneri Leonard Messel	Angiosperm	Asian	0	0	0.0833	0.0018	0.0009	-0.2949
99	Raemelton Farm	2011	Malus baccata Jackii	Angiosperm	Asian	0	0.4167	0	0.0018	0.0018	0.0426
100	Raemelton Farm	2011	Malus domestica Freedom	Angiosperm	unknown	0	0.4583	2.3542	0.0071	0.0096	0.1991
101	Raemelton Farm	2011	Malus domestica Liberty	Angiosperm	unknown	0	0.1944	1.5278	0.0107	0.0149	0.2134
102	Raemelton Farm	2011	Malus Donald Wyman	Angiosperm	unknown	0.0119	1.1786	2.0595	0.0124	0.0184	0.2405
103	Raemelton Farm	2011	Malus halliana Adirondack	Angiosperm	Asian	0.0238	0.3214	0.9167	0.0124	0.0175	0.2174
104	Raemelton Farm	2011	Malus Mary Potter	Angiosperm	Asian	0	1.2917	0.0417	0.0036	0.0035	0.0426
105	Raemelton Farm	2011	Malus Molten Lava	Angiosperm	unknown	0.0357	0.6667	1.7738	0.0124	0.0175	0.2174
106	Raemelton Farm	2011	Malus Pink Princess	Angiosperm	unknown	0.0208	0.1042	0.9792	0.0071	0.0088	0.153
107	Raemelton Farm	2011	Malus Prairifire	Angiosperm	unknown	0.037	0.7407	0.7222	0.016	0.0228	0.2227
108	Raemelton Farm	2011	Malus sargentii Select A	Angiosperm	Asian	0.0208	1.2708	0.5521	0.0142	0.0202	0.2204
109	Raemelton Farm	2011	Malus x zumi Calocarpa	Angiosperm	Asian	0.0238	1.7738	1.2262	0.0124	0.0184	0.2405
110	Raemelton Farm	2011	Metasequoia glyptostroboides	Gymnosperm	Asian	0	0.4167	0.8056	0.0053	0.0053	0.0426
111	Raemelton Farm	2011	Nyssa sylvatica	Angiosperm	Non Asian	0.0417	0.3333	0.5833	0.0036	0.0044	0.153
112	Raemelton Farm	2011	Nyssa sylvatica Tupelo Tower	Angiosperm	Non Asian	0	2	0.0833	0.0018	0.0026	0.2405
113	Raemelton Farm	2011	Nyssa sylvatica Wildfire	Angiosperm	Non Asian	0	0.25	1.25	0.0036	0.0053	0.2405
114	Raemelton Farm	2011	Oxydendrum arboreum	Angiosperm	Non Asian	0.1667	0	0.4167	0.0018	0.0026	0.2405
115	Raemelton Farm	2011	Parrotia persica	Angiosperm	Non Asian	0	0.3472	0.1389	0.0107	0.0053	-0.2949
116	Raemelton Farm	2011	Parrotia persica Vanessa	Angiosperm	Non Asian	0	0	1.1667	0.0018	0.0018	0.0426
117	Raemelton Farm	2011	Picea omorika	Gymnosperm	Non Asian	0	0	0.0859	0.0189	0.0061	-0.4735
118	Raemelton Farm	2011	Picea omorika Pendula	Gymnosperm	Non Asian	0	0	0	0.0036	0	-1
119	Raemelton Farm	2011	Picea orientalis Atrovirens	Gymnosperm	Non Asian	0	0	0	0.0018	0	-1
120	Raemelton Farm	2011	Picea orientalis Aurea Compacta	Gymnosperm	Non Asian	0	0	0.2917	0.0036	0.0026	-0.1009
121	Raemelton Farm	2011	Picea orientalis Gracillis	Gymnosperm	Non Asian	0	0	0.0417	0.0036	0.0009	-0.572
122	Raemelton Farm	2011	Picea pungens Fat Albert	Gymnosperm	Non Asian	0	0	0	0.0012	0	-1
123	Raemelton Farm	2011	Picea pungens Glauca	Gymnosperm	Non Asian	0	0	0	0.0024	0	-1
124	Raemelton Farm	2011	Picea pungens Glauca Fastigata	Gymnosperm	Non Asian	0	0	0.375	0.0012	0.0009	-0.1009
125	Raemelton Farm	2011	Picea pungens Glauca Majestic Blue	Gymnosperm	Non Asian	0	0	0	0.0018	0	-1
126	Raemelton Farm	2011	Picea pungens Glauca Van Sikes	Gymnosperm	Non Asian	0	0	0	0.013	0	-1

	Site	Year	Plant Taxon	Classification	Origin	Egg masses per 1 min survey	Nymphs per 1 min survey	Adults per 1 min survey	*p*	*r*	*E**
127	Raemelton Farm	2011	Picea pungens Hoopsii	Gymnosperm	Non Asian	0	0	0	0.0059	0	-1
128	Raemelton Farm	2011	Pinus cembra Silver Sheen	Gymnosperm	Non Asian	0	0.0833	2.8333	0.0018	0.0026	0.2405
129	Raemelton Farm	2011	Pinus flexilis Vanderwolf's Pyramid	Gymnosperm	Non Asian	0	0	0.875	0.0036	0.0053	0.2405
130	Raemelton Farm	2011	Pinus koraiensis Morris Blue	Gymnosperm	Asian	0	0	0	0.0036	0	-1
131	Raemelton Farm	2011	Pinus wallichiana	Gymnosperm	Asian	0	0	0.0833	0.0036	0.0018	-0.2949
132	Raemelton Farm	2011	Platanus x acerifolia Bloodgood	Angiosperm	Non Asian	0.0741	3.0185	1.2222	0.016	0.0202	0.1637
133	Raemelton Farm	2011	Platanus x acerifolia Yarwood	Angiosperm	Non Asian	0.0833	0.0833	0.0833	0.0018	0.0018	0.0426
134	Raemelton Farm	2011	Prunus cerasifera Cripoizam	Angiosperm	Non Asian	0.0278	0.1111	1.6944	0.0053	0.0053	0.0426
135	Raemelton Farm	2011	Prunus cerasifera Thundercloud	Angiosperm	Non Asian	0.0156	0	0.4375	0.0095	0.0044	-0.3241
136	Raemelton Farm	2011	Prunus cerasus Montmorency	Angiosperm	Non Asian	0	0.5833	0.5833	0.0018	0.0018	0.0426
137	Raemelton Farm	2011	Prunus mume	Angiosperm	Asian	0	0	0.0833	0.0018	0.0009	-0.2949
138	Raemelton Farm	2011	Prunus persica Red Haven	Angiosperm	Asian	0	0.2083	3.0625	0.0071	0.0105	0.2405
139	Raemelton Farm	2011	Prunus sargentii	Angiosperm	Asian	0	0.0556	0.1944	0.0053	0.0044	-0.0485
140	Raemelton Farm	2011	Prunus serrulata Kwanzan	Angiosperm	Asian	0.0278	2.4722	1.4444	0.0053	0.007	0.1843
141	Raemelton Farm	2011	Prunus serrulata Snowgoose	Angiosperm	Asian	0	0.0417	0.2917	0.0071	0.0044	-0.19
142	Raemelton Farm	2011	Prunus subhirtella Pendula	Angiosperm	Asian	0.0556	0.0833	0.6389	0.0053	0.0044	-0.0485
143	Raemelton Farm	2011	Prunus subhirtella Pisnshzam	Angiosperm	Asian	0	0	0	0.0018	0	-1
144	Raemelton Farm	2011	Prunus x incam Okame	Angiosperm	Asian	0	0.7083	0.5	0.0071	0.007	0.0426
145	Raemelton Farm	2011	Pseudocydonia sinensis	Angiosperm	Asian	0.0417	0.5417	0.25	0.0036	0.0053	0.2405
146	Raemelton Farm	2011	Pyrus communis Sunrise	Angiosperm	Non Asian	0	0.25	0.9167	0.0018	0.0018	0.0426
147	Raemelton Farm	2011	Pyrus fauriei Westwood	Angiosperm	Asian	0.0833	1	0.0833	0.0018	0.0026	0.2405
148	Raemelton Farm	2011	Quercus acutissima	Angiosperm	Asian	0	0	0.0833	0.0018	0.0009	-0.2949
149	Raemelton Farm	2011	Quercus alba	Angiosperm	Non Asian	0.0278	0.4444	0.6389	0.0053	0.0044	-0.0485
150	Raemelton Farm	2011	Quercus coccinea	Angiosperm	Non Asian	0	0.0556	0.1111	0.0107	0.0061	-0.2231
151	Raemelton Farm	2011	Quercus robur Regal Prince	Angiosperm	Non Asian	0.0882	2.6324	0.5147	0.0101	0.0131	0.1808
152	Raemelton Farm	2011	Quercus rubra	Angiosperm	Non Asian	0.0083	0.25	0.525	0.0178	0.0167	0.017
153	Raemelton Farm	2011	Rhus typhina Bailtiger	Angiosperm	Non Asian	0	0	0.2917	0.0036	0.0026	-0.1009
154	Raemelton Farm	2011	Sambucus nigra Eva	Angiosperm	Non Asian	0	0	0	0.0036	0	-1
155	Raemelton Farm	2011	Sophora japonica Regent	Angiosperm	Asian	0.0167	1.4083	1.225	0.0178	0.0228	0.1721
156	Raemelton Farm	2011	Stewartia koreana	Angiosperm	Asian	0	0.075	0.375	0.0059	0.0035	-0.2096
157	Raemelton Farm	2011	Stewartia pseudocamellia	Angiosperm	Asian	0.0357	0.2857	0.5357	0.0083	0.0105	0.1667
158	Raemelton Farm	2011	Styrax japonicus	Angiosperm	Asian	0.0185	0.3241	0.1111	0.016	0.0131	-0.0485
159	Raemelton Farm	2011	Syringa pekinensis Morton	Angiosperm	Asian	0	0.2667	13.9	0.0089	0.0131	0.2405
160	Raemelton Farm	2011	Syringa pekinensis Zhang Zhiming	Angiosperm	Asian	0.0278	0.7778	4.6944	0.0053	0.0079	0.2405
161	Raemelton Farm	2011	Taxus x media Hatfeldii	Gymnosperm	Hybrid	0	0	0.8333	0.0018	0.0018	0.0426
162	Raemelton Farm	2011	Taxus x media Hicksii	Gymnosperm	Hybrid	0	0	0.1667	0.0018	0.0009	-0.2949
163	Raemelton Farm	2011	Tetradium daniellii	Angiosperm	Asian	0.2083	1.5	1.5417	0.0071	0.0105	0.2405
164	Raemelton Farm	2011	Thuja plicata Emerald Cone	Gymnosperm	Non Asian	0	0	0	0.0018	0	-1
165	Raemelton Farm	2011	Thuja plicata Zebrina	Gymnosperm	Non Asian	0	0	0	0.0018	0	-1
166	Raemelton Farm	2011	Thuja standishii x plicata Green Giant	Gymnosperm	Hybrid	0	0	0.5833	0.0071	0.007	0.0426
167	Raemelton Farm	2011	Tilia cordata Greenspire	Angiosperm	Non Asian	0.0577	1.25	0.3654	0.0077	0.0096	0.1604
168	Raemelton Farm	2011	Tilia tomentosa Sterling	Angiosperm	Non Asian	0.125	1.8958	0.3958	0.0071	0.0096	0.1991

	Site	Year	Plant Taxon	Classification	Origin	Egg masses per 1 min survey	Nymphs per 1 min survey	Adults per 1 min survey	*p*	*r*	*E**
169	Raemelton Farm	2011	Ulmus americana Princeton	Angiosperm	Non Asian	0.0833	2.5119	0.8929	0.0124	0.0167	0.1929
170	Raemelton Farm	2011	Ulmus americana Valley Forge	Angiosperm	Non Asian	0.0972	2.9583	1.0278	0.0107	0.0158	0.2405
171	Raemelton Farm	2011	Ulmus parvifolia Emer I	Angiosperm	Asian	0	0.4444	0.1111	0.0053	0.0026	-0.2949
172	Raemelton Farm	2011	Ulmus parvifolia Emer II	Angiosperm	Asian	0	0.2708	0.625	0.0071	0.0079	0.1012
173	Raemelton Farm	2011	Viburnum carlesii Cayuga	Angiosperm	Asian	0	0	0.0833	0.0018	0.0009	-0.2949
174	Raemelton Farm	2011	Xanthoceras sorbifolia	Angiosperm	Asian	0	0	0.875	0.0024	0.0026	0.1012
175	Raemelton Farm	2012	Abies koreana	Gymnosperm	Asian	0	0	0	0.0159	0	-1
176	Raemelton Farm	2012	Abies nordmanniana	Gymnosperm	Non Asian	0	0	0	0.0027	0	-1
177	Raemelton Farm	2012	Acer campestre Evelyn	Angiosperm	Non Asian	0.0093	1.1759	0.0926	0.008	0.0165	0.4124
178	Raemelton Farm	2012	Acer griseum	Angiosperm	Asian	0.0062	0.3025	0.0617	0.0119	0.0082	-0.1104
179	Raemelton Farm	2012	Acer palmatum Bloodgood	Angiosperm	Asian	0	0.0139	0.0139	0.0053	0.0027	-0.2493
180	Raemelton Farm	2012	Acer palmatum Emperor I	Angiosperm	Asian	0	0	0	0.0027	0	-1
181	Raemelton Farm	2012	Acer palmatum Moonfire	Angiosperm	Asian	0	0	0	0.0027	0	-1
182	Raemelton Farm	2012	Acer palmatum Red Emperor	Angiosperm	Asian	0	0.0185	0	0.008	0.0027	-0.4279
183	Raemelton Farm	2012	Acer palmatum Sango Kaku	Angiosperm	Asian	0	0	0	0.0027	0	-1
184	Raemelton Farm	2012	Acer palmatum var. dissectum Inaba Shidare	Angiosperm	Asian	0	0	0	0.0053	0	-1
185	Raemelton Farm	2012	Acer palmatum var. dissectum Viridis	Angiosperm	Asian	0	0	0	0.0027	0	-1
186	Raemelton Farm	2012	Acer rubrum Bowhall	Angiosperm	Non Asian	0	0.9444	0	0.0066	0.0069	0.0917
187	Raemelton Farm	2012	Acer rubrum Brandywine	Angiosperm	Non Asian	0.0056	0.5944	0	0.0133	0.0247	0.3678
188	Raemelton Farm	2012	Acer rubrum Franksred	Angiosperm	Non Asian	0.0104	0.8576	0.0243	0.0212	0.0329	0.2864
189	Raemelton Farm	2012	Acer rubrum October Glory	Angiosperm	Non Asian	0.0278	0.6944	0.0278	0.0053	0.0096	0.3555
190	Raemelton Farm	2012	Acer rufinerve	Angiosperm	Asian	0	0.6111	0	0.0027	0.0027	0.0917
191	Raemelton Farm	2012	Acer saccharum Commemoration	Angiosperm	Non Asian	0.0556	0.5	0	0.0013	0.0041	0.5657
192	Raemelton Farm	2012	Acer saccharum Green Mountain	Angiosperm	Non Asian	0.0094	0.7956	0.022	0.0234	0.0302	0.1989
193	Raemelton Farm	2012	Acer saccharum Legacy	Angiosperm	Non Asian	0.0379	1.2424	0	0.0097	0.0165	0.3258
194	Raemelton Farm	2012	Acer truncatum	Angiosperm	Asian	0	0.0556	0	0.0013	0.0014	0.0917
195	Raemelton Farm	2012	Acer x freemanii Jeffersred	Angiosperm	Non Asian	0.0278	1.3611	0.0278	0.0027	0.0069	0.5006
196	Raemelton Farm	2012	Acer x tegmentosum White Tigress	Angiosperm	Asian	0	0	0	0.0027	0	-1
197	Raemelton Farm	2012	Aesculus x carnea Briotii	Angiosperm	Non Asian	0	0.0093	0	0.008	0.0014	-0.6662
198	Raemelton Farm	2012	Aesculus x carnea Fort McNair	Angiosperm	Non Asian	0	0.0741	0.0093	0.008	0.0055	-0.1104
199	Raemelton Farm	2012	Amelanchier x grandiflora Autumn Brilliance	Angiosperm	Non Asian	0	0.2593	0.4136	0.0119	0.0219	0.3624
200	Raemelton Farm	2012	Amelanchier x grandiflora Princess Diana	Angiosperm	Non Asian	0.0079	0.2063	0.1587	0.0093	0.0137	0.2639
201	Raemelton Farm	2012	Betula nigra BNMTF	Angiosperm	Non Asian	0	0	0.0278	0.0053	0.0027	-0.2493
202	Raemelton Farm	2012	Betula papyrifera Renci	Angiosperm	Non Asian	0	1.1111	0	0.0027	0.0027	0.0917
203	Raemelton Farm	2012	Calocedrus decurrens	Angiosperm	Non Asian	0	0.0278	0.1667	0.0027	0.0041	0.2864
204	Raemelton Farm	2012	Carpinus betulus Fastigiata	Angiosperm	Non Asian	0	0.2333	0.0111	0.0133	0.0041	-0.47
205	Raemelton Farm	2012	Carpinus betulus Frans Fontaine	Angiosperm	Non Asian	0	0	0.0648	0.008	0.0041	-0.2493
206	Raemelton Farm	2012	Carya illinoinensis Choctaw	Angiosperm	Non Asian	0	0.1111	0.1667	0.0013	0.0027	0.4124
207	Raemelton Farm	2012	Cedrus atlantica Glauca	Gymnosperm	Non Asian	0	0	0	0.004	0	-1
208	Raemelton Farm	2012	Cedrus deodara Karl Fuchs	Gymnosperm	Asian	0	0	0	0.0027	0	-1
209	Raemelton Farm	2012	Cedrus deodara Shalimar	Gymnosperm	Asian	0	0	0	0.0027	0	-1
210	Raemelton Farm	2012	Cercidiphyllum japonicum	Angiosperm	Asian	0	0.5926	0.0833	0.008	0.0096	0.1674

	Site	Year	Plant Taxon	Classification	Origin	Egg masses per 1 min survey	Nymphs per 1 min survey	Adults per 1 min survey	*p*	*r*	*E**
211	Raemelton Farm	2012	Cercis canadensis	Angiosperm	Non Asian	0.0694	3.1111	0.4583	0.0053	0.011	0.4124
212	Raemelton Farm	2012	Cercis canadensis Alba	Angiosperm	Non Asian	0	2.5278	0.0278	0.0027	0.0069	0.5006
213	Raemelton Farm	2012	Cercis canadensis Appalachian Red	Angiosperm	Non Asian	0.0417	0.2361	0.0139	0.0053	0.0096	0.3555
214	Raemelton Farm	2012	Cercis canadensis Covey	Angiosperm	Non Asian	0	0.8056	0.7222	0.0053	0.0096	0.3555
215	Raemelton Farm	2012	Cercis canadensis Forest Pansy	Angiosperm	Non Asian	0	0.1528	0.0139	0.0053	0.0082	0.2864
216	Raemelton Farm	2012	Cercis canadensis Pink Heartbreaker	Angiosperm	Non Asian	0	1.9722	0.0833	0.0027	0.0055	0.4124
217	Raemelton Farm	2012	Chamaecyparis nootkatensis Pendula	Gymnosperm	Non Asian	0	0	0	0.0106	0	-1
218	Raemelton Farm	2012	Chamaecyparis obtusa Aurea Nana	Gymnosperm	Asian	0	0	0	0.0013	0	-1
219	Raemelton Farm	2012	Chamaecyparis obtusa Compacta	Gymnosperm	Asian	0	0	0	0.0027	0	-1
220	Raemelton Farm	2012	Chamaecyparis obtusa Crippsii	Gymnosperm	Asian	0	0	0	0.0066	0	-1
221	Raemelton Farm	2012	Chionanthus retusus	Angiosperm	Asian	0	0	0	0.0053	0	-1
222	Raemelton Farm	2012	Cladrastis kentukea	Angiosperm	Non Asian	0.0303	1.0455	0.1465	0.0146	0.0329	0.4478
223	Raemelton Farm	2012	Cladrastis kentukea Perkins Pink	Angiosperm	Non Asian	0.0222	1.6556	0.0778	0.0066	0.0137	0.4124
224	Raemelton Farm	2012	Cornus controversa	Angiosperm	Asian	0	0.2222	0.75	0.0027	0.0082	0.5657
225	Raemelton Farm	2012	Cornus florida Appalachian Spring	Angiosperm	Non Asian	0	0	0.0556	0.0053	0.0027	-0.2493
226	Raemelton Farm	2012	Cornus florida Cherokee Princess	Angiosperm	Non Asian	0	0.1263	0.0051	0.0146	0.0069	-0.2934
227	Raemelton Farm	2012	Cornus florida Cloud 9	Angiosperm	Non Asian	0	0.2778	0.0694	0.0053	0.0027	-0.2493
228	Raemelton Farm	2012	Cornus florida COMCO #1	Angiosperm	Non Asian	0	0.0062	0.0309	0.0119	0.0055	-0.3037
229	Raemelton Farm	2012	Cornus florida Jean's Appalachian Snow	Angiosperm	Non Asian	0	0	0.0185	0.004	0.0014	-0.4279
230	Raemelton Farm	2012	Cornus florida Kay's Appalachian Mist	Angiosperm	Non Asian	0	0	0	0.004	0	-1
231	Raemelton Farm	2012	Cornus florida x kousa Aurora	Angiosperm	Hybrid	0	0	0	0.0027	0	-1
232	Raemelton Farm	2012	Cornus florida x kousa Constellation	Angiosperm	Hybrid	0	0	0	0.0027	0	-1
233	Raemelton Farm	2012	Cornus florida x kousa Ruth Ellen	Angiosperm	Hybrid	0	0	0.0556	0.0013	0.0014	0.0917
234	Raemelton Farm	2012	Cornus florida x kousa Stellar Pink	Angiosperm	Hybrid	0	0	0	0.004	0	-1
235	Raemelton Farm	2012	Cornus kousa Madison	Angiosperm	Asian	0	0	0	0.0022	0	-1
236	Raemelton Farm	2012	Cornus kousa National	Angiosperm	Asian	0	0	0	0.0027	0	-1
237	Raemelton Farm	2012	Cornus kousa Radiant Rose	Angiosperm	Asian	0	0	0	0.0004	0	-1
238	Raemelton Farm	2012	Cornus kousa Santomi	Angiosperm	Asian	0	0.0926	0.0417	0.0159	0.0082	-0.2493
239	Raemelton Farm	2012	Cornus kousa var chinensis Milky Way	Angiosperm	Asian	0	0.0139	0.0069	0.0106	0.0041	-0.3787
240	Raemelton Farm	2012	Cornus macrophylla	Angiosperm	Asian	0	0.4306	0.2917	0.0053	0.0123	0.4601
241	Raemelton Farm	2012	Cornus officinalis	Angiosperm	Asian	0	0	0.037	0.008	0.0027	-0.4279
242	Raemelton Farm	2012	Cornus walteri	Angiosperm	Non Asian	0	0	0	0.0027	0	-1
243	Raemelton Farm	2012	Crataegus laevigata Superba	Angiosperm	Non Asian	0	0	0	0.004	0	-1
244	Raemelton Farm	2012	Crataegus phaenopyrum	Angiosperm	Non Asian	0	0	0	0.0013	0	-1
245	Raemelton Farm	2012	Crataegus viridis Winter King	Angiosperm	Non Asian	0	0.0873	0.0079	0.0186	0.0069	-0.3994
246	Raemelton Farm	2012	Cryptomeria japonica Black Dragon	Gymnosperm	Asian	0	0	0	0.0027	0	-1
247	Raemelton Farm	2012	Cryptomeria japonica Gyokuryu	Gymnosperm	Asian	0	0	0	0.0027	0	-1
248	Raemelton Farm	2012	Cryptomeria japonica Yoshino	Gymnosperm	Asian	0	0	0	0.0027	0	-1
249	Raemelton Farm	2012	Ginkgo biloba Autumn Gold	Gymnosperm	Asian	0	0.1181	0.0139	0.0106	0.0027	-0.5379
250	Raemelton Farm	2012	Ginkgo biloba Magyar	Gymnosperm	Asian	0	0	0	0.008	0	-1
251	Raemelton Farm	2012	Ginkgo biloba Princeton Sentry	Gymnosperm	Asian	0	0	0.0278	0.0053	0.0027	-0.2493
252	Raemelton Farm	2012	Ginkgo biloba Saratoga	Gymnosperm	Asian	0	0	0	0.0027	0	-1

	Site	Year	Plant Taxon	Classification	Origin	Egg masses per 1 min survey	Nymphs per 1 min survey	Adults per 1 min survey	*p*	*r*	*E**
253	Raemelton Farm	2012	Gleditsia triacanthos Shademaster	Angiosperm	Non Asian	0.0046	0.412	0.1435	0.0159	0.0288	0.3555
254	Raemelton Farm	2012	Halesia tetraptera	Angiosperm	Non Asian	0	0.216	0.0556	0.0119	0.0151	0.1899
255	Raemelton Farm	2012	Hamamelis x intermedia Arnold Promise	Angiosperm	Hybrid	0	0	0	0.0035	0	-1
256	Raemelton Farm	2012	Hamamelis x intermedia Diane	Angiosperm	Hybrid	0	0.4	0.05	0.0044	0.0069	0.2864
257	Raemelton Farm	2012	Heptacodium miconioides	Angiosperm	Asian	0	0	0	0.0053	0	-1
258	Raemelton Farm	2012	Hibiscus syriacus Blue Bird	Angiosperm	Asian	0	0.0741	0.5	0.004	0.011	0.5244
259	Raemelton Farm	2012	Hibiscus syriacus Red Heart	Angiosperm	Asian	0	0	0.1667	0.0013	0.0027	0.4124
260	Raemelton Farm	2012	Ilex opaca Jersey Princess	Angiosperm	Non Asian	0	0.0278	0.0278	0.0027	0.0014	-0.2493
261	Raemelton Farm	2012	Juniperus chinensis Torulosa	Gymnosperm	Asian	0	0	0	0.0027	0	-1
262	Raemelton Farm	2012	Koelreuteria paniculata	Angiosperm	Asian	0.0128	0.5214	0.2436	0.0172	0.0288	0.3201
263	Raemelton Farm	2012	Liquidambar styraciflua	Angiosperm	Non Asian	0	1.1389	0.0278	0.0027	0.0041	0.2864
264	Raemelton Farm	2012	Liquidambar styraciflua Moraine	Angiosperm	Non Asian	0	0.0972	0.0139	0.0053	0.0055	0.0917
265	Raemelton Farm	2012	Liquidambar styraciflua Rotundiloba	Angiosperm	Non Asian	0	0	0	0.0027	0	-1
266	Raemelton Farm	2012	Magnolia liliiflora x stellata Ann	Angiosperm	Asian	0	0.0833	0	0.0027	0.0027	0.0917
267	Raemelton Farm	2012	Magnolia x loebneri Leonard Messel	Angiosperm	Asian	0	0.0278	0	0.0027	0.0014	-0.2493
268	Raemelton Farm	2012	Malus domestica Crimson Crisp	Angiosperm	unknown	0	0	0	0.0027	0	-1
269	Raemelton Farm	2012	Malus domestica Freedom	Angiosperm	unknown	0	0.0185	0.0741	0.004	0.0055	0.2315
270	Raemelton Farm	2012	Malus domestica Liberty	Angiosperm	unknown	0	0.0476	0.0397	0.0093	0.0082	0.0149
271	Raemelton Farm	2012	Malus Donald Wyman	Angiosperm	unknown	0	0.7014	0.5208	0.0106	0.0274	0.5006
272	Raemelton Farm	2012	Malus halliana Adirondack	Angiosperm	Asian	0	0.0833	0.3889	0.008	0.0192	0.4743
273	Raemelton Farm	2012	Malus Molten Lava	Angiosperm	unknown	0	0.3519	0.8611	0.008	0.0219	0.5244
274	Raemelton Farm	2012	Malus Pink Princess	Angiosperm	unknown	0	0.1481	0.0648	0.008	0.011	0.2315
275	Raemelton Farm	2012	Malus Prairifire	Angiosperm	unknown	0.0694	0.2685	0.588	0.0159	0.0425	0.5128
276	Raemelton Farm	2012	Malus sargentii Select A	Angiosperm	Asian	0.0056	0.05	0.1278	0.0133	0.0178	0.2195
277	Raemelton Farm	2012	Malus x zumi Calocarpa	Angiosperm	Asian	0.0185	0.0926	0.1389	0.008	0.0151	0.3757
278	Raemelton Farm	2012	Metasequoia glyptostroboides	Gymnosperm	Asian	0	0	0	0.0027	0	-1
279	Raemelton Farm	2012	Nyssa sylvatica	Angiosperm	Non Asian	0	0.7778	0	0.0053	0.0041	-0.0519
280	Raemelton Farm	2012	Nyssa sylvatica Tupelo Tower	Angiosperm	Non Asian	0	0.75	0	0.0027	0.0027	0.0917
281	Raemelton Farm	2012	Nyssa sylvatica Wildfire	Angiosperm	Non Asian	0	0	0.0278	0.0027	0.0014	-0.2493
282	Raemelton Farm	2012	Oxydendrum arboreum	Angiosperm	Non Asian	0	0.1111	0	0.0027	0.0041	0.2864
283	Raemelton Farm	2012	Parrotia persica	Angiosperm	Non Asian	0	0	0.0278	0.0027	0.0014	-0.2493
284	Raemelton Farm	2012	Parrotia persica Ruby Vase	Angiosperm	Non Asian	0	0	0	0.0027	0	-1
285	Raemelton Farm	2012	Picea omorika	Gymnosperm	Non Asian	0	0	0	0.0053	0	-1
286	Raemelton Farm	2012	Picea omorika Pendula	Gymnosperm	Non Asian	0	0	0	0.0013	0	-1
287	Raemelton Farm	2012	Picea orientalis Atrovirens	Gymnosperm	Non Asian	0	0	0	0.0027	0	-1
288	Raemelton Farm	2012	Picea orientalis Aurea Compacta	Gymnosperm	Non Asian	0	0	0	0.0013	0	-1
289	Raemelton Farm	2012	Picea orientalis Gracillis	Gymnosperm	Non Asian	0	0	0	0.0013	0	-1
290	Raemelton Farm	2012	Picea pungens Hoopsii	Gymnosperm	Non Asian	0	0	0	0.0027	0	-1
291	Raemelton Farm	2012	Pinus bungeana	Gymnosperm	Asian	0	0	0	0.0027	0	-1
292	Raemelton Farm	2012	Pinus flexilis Vanderwolf's Pyramid	Gymnosperm	Non Asian	0	0	0.0139	0.0053	0.0014	-0.5379
293	Raemelton Farm	2012	Pinus koraiensis	Gymnosperm	Asian	0	0	0	0.0027	0	-1
294	Raemelton Farm	2012	Pinus koraiensis Morris Blue	Gymnosperm	Asian	0	0	0	0.0027	0	-1

	Site	Year	Plant Taxon	Classification	Origin	Egg masses per 1 min survey	Nymphs per 1 min survey	Adults per 1 min survey	*p*	*r*	*E**
295	Raemelton Farm	2012	Pinus parvifolia	Gymnosperm	Asian	0	0	0	0.0027	0	-1
296	Raemelton Farm	2012	Pinus strobus Pendula	Gymnosperm	Non Asian	0	0	0	0.0027	0	-1
297	Raemelton Farm	2012	Pinus thunbergii Thunderhead	Gymnosperm	Asian	0	0	0	0.0013	0	-1
298	Raemelton Farm	2012	Platanus x acerifolia Bloodgood	Angiosperm	Non Asian	0	0.1597	0.0347	0.0106	0.0069	-0.1421
299	Raemelton Farm	2012	Platanus x acerifolia Yarwood	Angiosperm	Non Asian	0	0.6296	0.0556	0.008	0.0055	-0.1104
300	Raemelton Farm	2012	Prunus avium BaDa Bing	Angiosperm	Non Asian	0	0.0556	0	0.0027	0.0027	0.0917
301	Raemelton Farm	2012	Prunus avium Stella	Angiosperm	Non Asian	0	1.1111	0.0556	0.0013	0.0027	0.4124
302	Raemelton Farm	2012	Prunus cerasifera Cripoizam	Angiosperm	Non Asian	0	0.0238	0.0794	0.0093	0.011	0.1574
303	Raemelton Farm	2012	Prunus cerasifera Thundercloud	Angiosperm	Non Asian	0	0.0139	0	0.0053	0.0014	-0.5379
304	Raemelton Farm	2012	Prunus cerasus	Angiosperm	Non Asian	0	0	0	0.0027	0	-1
305	Raemelton Farm	2012	Prunus cerasus Montmorency	Angiosperm	Non Asian	0	1.1111	0	0.0013	0.0014	0.0917
306	Raemelton Farm	2012	Prunus persica Red Haven	Angiosperm	Asian	0	0.3333	0.4889	0.0066	0.0137	0.4124
307	Raemelton Farm	2012	Prunus sargentii	Angiosperm	Asian	0	0.0278	0.0093	0.008	0.0041	-0.2493
308	Raemelton Farm	2012	Prunus serrula Tibetica	Angiosperm	Asian	0	0	0	0.0013	0	-1
309	Raemelton Farm	2012	Prunus serrulata Kwanzan	Angiosperm	Asian	0.0111	0.5667	0.0333	0.0066	0.0137	0.4124
310	Raemelton Farm	2012	Prunus serrulata Snowgoose	Angiosperm	Asian	0	0.6759	0.1852	0.008	0.0123	0.2864
311	Raemelton Farm	2012	Prunus subhirtella Pendula	Angiosperm	Asian	0	0.2222	0.2315	0.008	0.011	0.2315
312	Raemelton Farm	2012	Prunus subhirtella Pisnshzam	Angiosperm	Asian	0	0.0139	0.2083	0.0053	0.0082	0.2864
313	Raemelton Farm	2012	Prunus x incam Okame	Angiosperm	Asian	0.0093	0.0185	0.0093	0.008	0.0027	-0.4279
314	Raemelton Farm	2012	Prunus x yedoensis	Angiosperm	Asian	0	0.5556	0	0.0013	0.0014	0.0917
315	Raemelton Farm	2012	Pseudocydonia sinensis	Angiosperm	Asian	0	0	0	0.0027	0	-1
316	Raemelton Farm	2012	Pyrus betulifolia	Angiosperm	Asian	0	0.0278	0	0.0027	0.0014	-0.2493
317	Raemelton Farm	2012	Pyrus communis Blake's Pride	Angiosperm	Non Asian	0	0	0	0.0013	0	-1
318	Raemelton Farm	2012	Pyrus communis Sunrise	Angiosperm	Non Asian	0	0.0556	0	0.0013	0.0014	0.0917
319	Raemelton Farm	2012	Pyrus fauriei Westwood	Angiosperm	Asian	0	0.4444	0	0.0013	0.0027	0.4124
320	Raemelton Farm	2012	Quercus acutissima	Angiosperm	Asian	0	1.1111	0.1111	0.0027	0.0041	0.2864
321	Raemelton Farm	2012	Quercus alba	Angiosperm	Non Asian	0	0.4028	0.0139	0.0053	0.0041	-0.0519
322	Raemelton Farm	2012	Quercus coccinea	Angiosperm	Non Asian	0	0.2121	0.0152	0.0146	0.0055	-0.3918
323	Raemelton Farm	2012	Quercus robur Fastigiata	Angiosperm	Non Asian	0	0	0.0556	0.0027	0.0014	-0.2493
324	Raemelton Farm	2012	Quercus robur Regal Prince	Angiosperm	Non Asian	0.0247	0.679	0.0494	0.0119	0.0137	0.1436
325	Raemelton Farm	2012	Quercus rubra	Angiosperm	Non Asian	0.0035	0.684	0.0035	0.0212	0.011	-0.2493
326	Raemelton Farm	2012	Rhus typhina Bailtiger	Angiosperm	Non Asian	0	0	0	0.0027	0	-1
327	Raemelton Farm	2012	Sambucus nigra Eva	Angiosperm	Non Asian	0	0	0	0.0027	0	-1
328	Raemelton Farm	2012	Sophora japonica Millstone	Angiosperm	Asian	0	0.6944	0.2222	0.0027	0.0082	0.5657
329	Raemelton Farm	2012	Sophora japonica Regent	Angiosperm	Asian	0.0171	0.4786	0.0812	0.0172	0.0316	0.3603
330	Raemelton Farm	2012	Stewartia koreana	Angiosperm	Asian	0	0	0.0278	0.0027	0.0014	-0.2493
331	Raemelton Farm	2012	Stewartia pseudocamellia	Angiosperm	Asian	0	0.0741	0.213	0.008	0.0178	0.4451
332	Raemelton Farm	2012	Styrax japonicus	Angiosperm	Asian	0	0.1061	0.0051	0.0146	0.0041	-0.5063
333	Raemelton Farm	2012	Styrax obassia	Angiosperm	Asian	0	0.4167	0.0278	0.0027	0.0041	0.2864
334	Raemelton Farm	2012	Syringa pekinensis Morton	Angiosperm	Asian	0.0093	0.5648	0.5	0.008	0.0247	0.5657
335	Raemelton Farm	2012	Syringa pekinensis Zhang Zhiming	Angiosperm	Asian	0	0.3889	6.9722	0.0027	0.0082	0.5657
336	Raemelton Farm	2012	Tetradium daniellii	Angiosperm	Asian	0.0139	1.625	0.1111	0.0053	0.0137	0.5006

	Site	Year	Plant Taxon	Classification	Origin	Egg masses per 1 min survey	Nymphs per 1 min survey	Adults per 1 min survey	*p*	*r*	*E**
337	Raemelton Farm	2012	Thuja plicata Atrovirens	Gymnosperm	Non Asian	0.0139	0	0.0417	0.0053	0.0041	-0.0519
338	Raemelton Farm	2012	Thuja plicata Emerald Cone	Gymnosperm	Non Asian	0	0	0	0.0027	0	-1
339	Raemelton Farm	2012	Thuja standishii x plicata Green Giant	Gymnosperm	Hybrid	0	0	0	0.0053	0	-1
340	Raemelton Farm	2012	Tilia cordata Greenspire	Angiosperm	Non Asian	0.0093	0.463	0.0278	0.008	0.011	0.2315
341	Raemelton Farm	2012	Tilia tomentosa Sterling	Angiosperm	Non Asian	0.0278	0.6204	0.0185	0.008	0.0082	0.0917
342	Raemelton Farm	2012	Tsuga canadensis Pendula	Gymnosperm	Non Asian	0	0	0	0.0013	0	-1
343	Raemelton Farm	2012	Ulmus americana Princeton	Angiosperm	Non Asian	0.0093	0.6111	0.0093	0.008	0.0137	0.334
344	Raemelton Farm	2012	Ulmus americana Valley Forge	Angiosperm	Non Asian	0	0.1667	0	0.0027	0.0027	0.0917
345	Raemelton Farm	2012	Ulmus parvifolia Emer II	Angiosperm	Asian	0	0	0.0093	0.008	0.0014	-0.6662
346	Raemelton Farm	2012	Xanthoceras sorbifolia	Angiosperm	Asian	0	0.213	0	0.008	0.0041	-0.2493
347	Ruppert East	2012	Acer rubrum October Glory	Angiosperm	Non Asian	0.0069	1.0069	0	0.0625	0.0989	0.256
348	Ruppert East	2012	Acer saccharum Commemoration	Angiosperm	Non Asian	0	0.0278	0	0.0156	0.0055	-0.4544
349	Ruppert East	2012	Acer saccharum Green Mountain	Angiosperm	Non Asian	0.0159	0.9643	0.2143	0.1094	0.1484	0.1827
350	Ruppert East	2012	Acer saccharum Legacy	Angiosperm	Non Asian	0	0.2639	0	0.0313	0.0275	-0.0321
351	Ruppert East	2012	Amelanchier x grandiflora Autumn Brilliance	Angiosperm	Non Asian	0.0324	1.1111	0.0046	0.0938	0.1593	0.2891
352	Ruppert East	2012	Betula nigra Heritage	Angiosperm	Non Asian	0	0.1875	0	0.0208	0.0165	-0.0846
353	Ruppert East	2012	Carpinus betulus Fastigiata	Angiosperm	Non Asian	0.0208	0.375	0.0069	0.0625	0.0549	-0.0321
354	Ruppert East	2012	Cercis canadensis	Angiosperm	Non Asian	0.0556	2.6528	0	0.0313	0.0604	0.3471
355	Ruppert East	2012	Crataegus viridis Winter King	Angiosperm	Non Asian	0	0.0417	0	0.0313	0.011	-0.4544
356	Ruppert East	2012	Gleditsia triacanthos Shademaster	Angiosperm	Non Asian	0	0.5606	0.1894	0.0573	0.0879	0.2416
357	Ruppert East	2012	Malus Adams	Angiosperm	unknown	0	0.6528	0.0417	0.0313	0.033	0.059
358	Ruppert East	2012	Malus Prairifire	Angiosperm	unknown	0.0417	0.4861	0.0139	0.0313	0.044	0.2002
359	Ruppert East	2012	Malus Spring Snow	Angiosperm	unknown	0	0.1111	0	0.0156	0.0055	-0.4544
360	Ruppert East	2012	Malus x zumi Calocarpa	Angiosperm	Asian	0	0.9861	0	0.0313	0.0275	-0.0321
361	Ruppert East	2012	Picea abies	Gymnosperm	Non Asian	0	0.0069	0.0139	0.0625	0.0165	-0.5608
362	Ruppert East	2012	Prunus serrulata Kwanzan	Angiosperm	Asian	0.0139	0.3611	0	0.0313	0.022	-0.1427
363	Ruppert East	2012	Quercus acutissima	Angiosperm	Asian	0.0139	0.4306	0.0278	0.0313	0.0549	0.3045
364	Ruppert East	2012	Thuja occidentalis Smaragd	Gymnosperm	Non Asian	0	0.197	0	0.0286	0.022	-0.0998
365	Ruppert East	2012	Thuja standishii x plicata Green Giant	Gymnosperm	Hybrid	0	0.0067	0	0.0651	0.0055	-0.8348
366	Ruppert East	2012	Tilia cordata Greenspire	Angiosperm	Non Asian	0.0556	0	0	0.0156	0.011	-0.1427
367	Ruppert East	2012	Ulmus parvifolia Dynasty	Angiosperm	Asian	0	0.6111	0	0.0156	0.011	-0.1427
368	Ruppert East	2012	Ulmus parvifolia Emer II	Angiosperm	Asian	0	0.125	0.0139	0.0313	0.033	0.059
369	Ruppert East	2012	Zelkova serrata Village Green	Angiosperm	Asian	0	0.2407	0.0093	0.0938	0.044	-0.3332
370	Ruppert North	2012	Acer rubrum Armstrong	Angiosperm	Non Asian	0	2.4352	0.0093	0.039	0.0662	0.2614
371	Ruppert North	2012	Acer rubrum Franksred	Angiosperm	Non Asian	0.0069	1.0023	0.0116	0.1558	0.1397	-0.0519
372	Ruppert North	2012	Acer rubrum October Glory	Angiosperm	Non Asian	0.0115	1.6897	0.023	0.0628	0.1066	0.2614
373	Ruppert North	2012	Acer rubrum Sun Valley	Angiosperm	Non Asian	0	1.6111	0	0.013	0.0147	0.0648
374	Ruppert North	2012	Acer saccharum Commemoration	Angiosperm	Non Asian	0	1.3056	0.0833	0.013	0.0221	0.2614
375	Ruppert North	2012	Acer saccharum Green Mountain	Angiosperm	Non Asian	0.0208	0.4028	0.0208	0.0519	0.0699	0.1497
376	Ruppert North	2012	Betula nigra BNMTF	Angiosperm	Non Asian	0	0.4583	0.0278	0.026	0.0294	0.0648
377	Ruppert North	2012	Crataegus viridis Winter King	Angiosperm	Non Asian	0.0093	0.5093	0.0278	0.039	0.0441	0.0648
378	Ruppert North	2012	Cryptomeria japonica	Gymnosperm	Asian	0	0.5833	0.0556	0.013	0.011	-0.0788

	Site	Year	Plant Taxon	Classification	Origin	Egg masses per 1 min survey	Nymphs per 1 min survey	Adults per 1 min survey	*p*	*r*	*E**
379	Ruppert North	2012	Cupressocyparis leylandii	Gymnosperm	Non Asian	0	0.4375	0	0.0173	0.0074	-0.4016
380	Ruppert North	2012	Gleditsia triacanthos Shademaster	Angiosperm	Non Asian	0.0056	0.55	0.0444	0.0649	0.0772	0.089
381	Ruppert North	2012	Gleditsia triacanthos Skyline	Angiosperm	Non Asian	0	0.4352	0.0648	0.039	0.0404	0.0214
382	Ruppert North	2012	Ilex x aquipernyi Meschick	Angiosperm	Hybrid	0	0.1444	0.0056	0.0649	0.0184	-0.5569
383	Ruppert North	2012	Malus Prairifire	Angiosperm	unknown	0	0.7222	0	0.013	0.0147	0.0648
384	Ruppert North	2012	Ostrya virginiana	Angiosperm	Non Asian	0	0.2778	0.0278	0.013	0.0074	-0.2745
385	Ruppert North	2012	Picea abies	Gymnosperm	Non Asian	0	0.2222	0	0.013	0.0037	-0.5569
386	Ruppert North	2012	Platanus x acerifolia Bloodgood	Angiosperm	Non Asian	0.0083	0.3778	0.0583	0.1299	0.136	0.0259
387	Ruppert North	2012	Prunus sargentii	Angiosperm	Asian	0	0.1944	0	0.013	0.011	-0.0788
388	Ruppert North	2012	Prunus serrulata Kwanzan	Angiosperm	Asian	0.0139	0.4722	0.0556	0.026	0.0147	-0.2745
389	Ruppert North	2012	Prunus x incam Okame	Angiosperm	Asian	0	0.3056	0.0139	0.026	0.011	-0.4016
390	Ruppert North	2012	Prunus x yedoensis	Angiosperm	Asian	0	0.2222	0.0069	0.0519	0.0184	-0.4751
391	Ruppert North	2012	Syringa reticulata Ivory Silk	Angiosperm	Asian	0	1.7778	0.1389	0.013	0.0221	0.2614
392	Ruppert North	2012	Thuja standishii x plicata Green Giant	Gymnosperm	Hybrid	0	0.0333	0.1	0.0216	0.0257	0.089
393	Ruppert North	2012	Tilia cordata Greenspire	Angiosperm	Non Asian	0.0278	0.3148	0	0.039	0.0368	-0.0263
394	Ruppert North	2012	Tilia tomentosa Sterling	Angiosperm	Non Asian	0.0385	1.0641	0.0128	0.0281	0.0404	0.182
395	Ruppert North	2012	Ulmus parvifolia Dynasty	Angiosperm	Asian	0	0.4167	0	0.013	0.011	-0.0788
396	Raemelton Farm	2013	Abies koreana	Gymnosperm	Asian	0	0	0.0043	0.0151	0.001	-0.8674
397	Raemelton Farm	2013	Abies nordmanniana	Gymnosperm	Non Asian	0	0	0	0.0023	0	-1
398	Raemelton Farm	2013	Acer campestre Evelyn	Angiosperm	Non Asian	0.0185	0.5926	0.0278	0.007	0.0078	0.1035
399	Raemelton Farm	2013	Acer griseum	Angiosperm	Asian	0	0.0185	0.0062	0.0105	0.0019	-0.6595
400	Raemelton Farm	2013	Acer palmatum Bloodgood	Angiosperm	Asian	0.0093	0.0093	0.0463	0.007	0.0039	-0.238
401	Raemelton Farm	2013	Acer palmatum Emperor I	Angiosperm	Asian	0	0	0	0.0023	0	-1
402	Raemelton Farm	2013	Acer palmatum Red Emperor	Angiosperm	Asian	0	0.0093	0	0.007	0.001	-0.7333
403	Raemelton Farm	2013	Acer palmatum Sango Kaku	Angiosperm	Asian	0	0	0	0.0023	0	-1
404	Raemelton Farm	2013	Acer palmatum var. dissectum Inaba Shidare	Angiosperm	Asian	0	0	0	0.0047	0	-1
405	Raemelton Farm	2013	Acer palmatum var. dissectum Viridis	Angiosperm	Asian	0	0	0	0.0023	0	-1
406	Raemelton Farm	2013	Acer rubrum Bowhall	Angiosperm	Non Asian	0	1.0556	0.0333	0.0058	0.0078	0.1926
407	Raemelton Farm	2013	Acer rubrum Brandywine	Angiosperm	Non Asian	0.0389	1.6889	0.1444	0.0117	0.0243	0.3954
408	Raemelton Farm	2013	Acer rubrum Franksred	Angiosperm	Non Asian	0.0299	1.9872	0.0769	0.0151	0.0272	0.3308
409	Raemelton Farm	2013	Acer rubrum October Glory	Angiosperm	Non Asian	0.0556	1.25	0.0278	0.0047	0.0078	0.2974
410	Raemelton Farm	2013	Acer rufinerve	Angiosperm	Asian	0	1.3056	0	0.0023	0.001	-0.3684
411	Raemelton Farm	2013	Acer saccharum Commemoration	Angiosperm	Non Asian	0	0.3333	0.2778	0.0012	0.0029	0.4694
412	Raemelton Farm	2013	Acer saccharum Green Mountain	Angiosperm	Non Asian	0.0226	1.2062	0.113	0.0229	0.033	0.2296
413	Raemelton Farm	2013	Acer saccharum Legacy	Angiosperm	Non Asian	0.0625	1.5208	0.1563	0.0062	0.0107	0.3113
414	Raemelton Farm	2013	Acer truncatum	Angiosperm	Asian	0	0	0	0.0012	0	-1
415	Raemelton Farm	2013	Acer x freemanii Jeffersred	Angiosperm	Non Asian	0	6.7778	0.0556	0.0012	0.0029	0.4694
416	Raemelton Farm	2013	Acer x tegmentosum White Tigress	Angiosperm	Asian	0	0	0	0.0023	0	-1
417	Raemelton Farm	2013	Aesculus x carnea Briotii	Angiosperm	Non Asian	0	0.0278	0	0.007	0.0029	-0.3684
418	Raemelton Farm	2013	Aesculus x carnea Fort McNair	Angiosperm	Non Asian	0.0278	0.4074	0	0.007	0.0029	-0.3684
419	Raemelton Farm	2013	Amelanchier x grandiflora Autumn Brilliance	Angiosperm	Non Asian	0	0.3232	0.6111	0.0128	0.0262	0.3876
420	Raemelton Farm	2013	Amelanchier x grandiflora Princess Diana	Angiosperm	Non Asian	0.0079	0.254	0.119	0.0082	0.0146	0.3285

	Site	Year	Plant Taxon	Classification	Origin	Egg masses per 1 min survey	Nymphs per 1 min survey	Adults per 1 min survey	*p*	*r*	*E**
421	Raemelton Farm	2013	Betula nigra BNMTF	Angiosperm	Non Asian	0.0278	0.4861	0	0.0047	0.0039	-0.0399
422	Raemelton Farm	2013	Betula nigra Cully	Angiosperm	Non Asian	0	0	0.0278	0.0023	0.001	-0.3684
423	Raemelton Farm	2013	Betula papyrifera Renci	Angiosperm	Non Asian	0	0	0	0.0023	0	-1
424	Raemelton Farm	2013	Calocedrus decurrens	Angiosperm	Non Asian	0	0.0278	0.0278	0.0047	0.0029	-0.1817
425	Raemelton Farm	2013	Carpinus betulus Fastigiata	Angiosperm	Non Asian	0.0062	0.3765	0.0247	0.0105	0.0097	0.0127
426	Raemelton Farm	2013	Carpinus betulus Frans Fontaine	Angiosperm	Non Asian	0	0.3194	0.0139	0.0047	0.0019	-0.3684
427	Raemelton Farm	2013	Carya illinoinensis Choctaw	Angiosperm	Non Asian	0	0.7778	0.3889	0.0012	0.0029	0.4694
428	Raemelton Farm	2013	Cedrus atlantica Glauca	Gymnosperm	Non Asian	0	0	0	0.007	0	-1
429	Raemelton Farm	2013	Cedrus atlantica Kroh's Twisted	Gymnosperm	Non Asian	0	0	0	0.0012	0	-1
430	Raemelton Farm	2013	Cedrus deodara Karl Fuchs	Gymnosperm	Asian	0	0	0	0.0023	0	-1
431	Raemelton Farm	2013	Cedrus deodara Shalimar	Gymnosperm	Asian	0	0.1111	0	0.0023	0.001	-0.3684
432	Raemelton Farm	2013	Cercidiphyllum japonicum	Angiosperm	Asian	0	0.825	0.325	0.0078	0.0146	0.3501
433	Raemelton Farm	2013	Cercis canadensis	Angiosperm	Non Asian	0.0139	1.3472	0.0417	0.0047	0.0068	0.2354
434	Raemelton Farm	2013	Cercis canadensis Alba	Angiosperm	Non Asian	0	0.3889	0.3889	0.0023	0.0049	0.3954
435	Raemelton Farm	2013	Cercis canadensis Appalachian Red	Angiosperm	Non Asian	0	0.6806	0.0417	0.0047	0.0058	0.1614
436	Raemelton Farm	2013	Cercis canadensis Covey	Angiosperm	Non Asian	0.0556	0	0	0.0035	0.001	-0.5294
437	Raemelton Farm	2013	Cercis canadensis Forest Pansy	Angiosperm	Non Asian	0	1	0.0278	0.0023	0.0039	0.2974
438	Raemelton Farm	2013	Cercis canadensis Pink Heartbreaker	Angiosperm	Non Asian	0	0	0	0.0023	0	-1
439	Raemelton Farm	2013	Chamaecyparis nootkatensis Pendula	Gymnosperm	Non Asian	0	0	0.0139	0.014	0.001	-0.8571
440	Raemelton Farm	2013	Chamaecyparis nootkatensis Pendula Glauca	Gymnosperm	Non Asian	0	0.0278	0	0.0023	0.001	-0.3684
441	Raemelton Farm	2013	Chamaecyparis obtusa Aurea Nana	Gymnosperm	Asian	0	0	0	0.0016	0	-1
442	Raemelton Farm	2013	Chamaecyparis obtusa Compacta	Gymnosperm	Asian	0	0	0	0.0019	0	-1
443	Raemelton Farm	2013	Chamaecyparis obtusa Crippsii	Gymnosperm	Asian	0	0	0.0079	0.0082	0.001	-0.767
444	Raemelton Farm	2013	Chamaecyparis obtusa Gimborn's Beauty	Gymnosperm	Asian	0	0	0	0.0012	0	-1
445	Raemelton Farm	2013	Chamaecyparis obtusa Kosteri	Gymnosperm	Asian	0	0	0	0.0012	0	-1
446	Raemelton Farm	2013	Chionanthus retusus	Angiosperm	Asian	0	0.0694	0.0833	0.0047	0.0039	-0.0399
447	Raemelton Farm	2013	Cladrastis kentukea	Angiosperm	Non Asian	0.0079	0.5873	0.2302	0.0082	0.0126	0.2632
448	Raemelton Farm	2013	Cladrastis kentukea Perkins Pink	Angiosperm	Non Asian	0	0.9028	0.125	0.0047	0.0078	0.2974
449	Raemelton Farm	2013	Cornus controversa	Angiosperm	Asian	0	0.1389	0.1528	0.0047	0.0078	0.2974
450	Raemelton Farm	2013	Cornus florida Appalachian Spring	Angiosperm	Non Asian	0	0.5	0.0139	0.0047	0.0019	-0.3684
451	Raemelton Farm	2013	Cornus florida Cherokee Princess	Angiosperm	Non Asian	0	0.0741	0	0.0105	0.0029	-0.5294
452	Raemelton Farm	2013	Cornus florida Cloud 9	Angiosperm	Non Asian	0	0.0556	0.0139	0.0047	0.0039	-0.0399
453	Raemelton Farm	2013	Cornus florida COMCO #1	Angiosperm	Non Asian	0	0.0864	0.0309	0.0105	0.0049	-0.322
454	Raemelton Farm	2013	Cornus florida Jean's Appalachian Snow	Angiosperm	Non Asian	0	0	0.1667	0.0035	0.0029	-0.0399
455	Raemelton Farm	2013	Cornus florida Kay's Appalachian Mist	Angiosperm	Non Asian	0	0.037	0.0556	0.0035	0.0029	-0.0399
456	Raemelton Farm	2013	Cornus florida x kousa Aurora	Angiosperm	Hybrid	0	0.0278	0	0.0023	0.001	-0.3684
457	Raemelton Farm	2013	Cornus florida x kousa Constellation	Angiosperm	Hybrid	0	0.1111	0	0.0012	0.0019	0.2974
458	Raemelton Farm	2013	Cornus florida x kousa Ruth Ellen	Angiosperm	Hybrid	0	1.2222	0.1667	0.0012	0.0019	0.2974
459	Raemelton Farm	2013	Cornus florida x kousa Stellar Pink	Angiosperm	Hybrid	0	0.0278	0	0.0023	0.001	-0.3684
460	Raemelton Farm	2013	Cornus kousa Madison	Angiosperm	Asian	0	0.0667	0.0667	0.0019	0.0019	0.0512
461	Raemelton Farm	2013	Cornus kousa National	Angiosperm	Asian	0	0.4444	0	0.0023	0.0049	0.3954
462	Raemelton Farm	2013	Cornus kousa Radiant Rose	Angiosperm	Asian	0	0	0	0.0004	0	-1

	Site	Year	Plant Taxon	Classification	Origin	Egg masses per 1 min survey	Nymphs per 1 min survey	Adults per 1 min survey	*p*	*r*	*E**
463	Raemelton Farm	2013	Cornus kousa Santomi	Angiosperm	Asian	0	0.2824	0.037	0.014	0.0117	-0.0399
464	Raemelton Farm	2013	Cornus kousa var chinensis Milky Way	Angiosperm	Asian	0	0.0278	0.1481	0.007	0.0087	0.1614
465	Raemelton Farm	2013	Cornus macrophylla	Angiosperm	Asian	0.0139	0.5139	0.0556	0.0047	0.0058	0.1614
466	Raemelton Farm	2013	Cornus officinalis	Angiosperm	Asian	0	0	0.0833	0.007	0.0029	-0.3684
467	Raemelton Farm	2013	Cornus walteri	Angiosperm	Non Asian	0	0.8333	0.0833	0.0023	0.0039	0.2974
468	Raemelton Farm	2013	Crataegus laevigata Superba	Angiosperm	Non Asian	0	0	0	0.0035	0	-1
469	Raemelton Farm	2013	Crataegus phaenopyrum	Angiosperm	Non Asian	0	0.0185	0.037	0.0035	0.0019	-0.238
470	Raemelton Farm	2013	Crataegus viridis Winter King	Angiosperm	Non Asian	0.0079	0.1746	0.1071	0.0163	0.0146	-0.0054
471	Raemelton Farm	2013	Cryptomeria japonica Black Dragon	Gymnosperm	Asian	0	0	0	0.0058	0	-1
472	Raemelton Farm	2013	Cryptomeria japonica Gyokuryu	Gymnosperm	Asian	0	0	0	0.007	0	-1
473	Raemelton Farm	2013	Cryptomeria japonica Yoshino	Gymnosperm	Asian	0	0.4	0.1111	0.0058	0.0029	-0.2871
474	Raemelton Farm	2013	Ficus carica Chicago Hardy	Angiosperm	Non Asian	0.1111	0.1667	0.1111	0.0012	0.0019	0.2974
475	Raemelton Farm	2013	Ginkgo biloba Autumn Gold	Gymnosperm	Asian	0.0159	0.0079	0.0317	0.0082	0.0049	-0.2052
476	Raemelton Farm	2013	Ginkgo biloba Magyar	Gymnosperm	Asian	0	0	0.0185	0.007	0.001	-0.7333
477	Raemelton Farm	2013	Ginkgo biloba Princeton Sentry	Gymnosperm	Asian	0.0159	0.3571	0.0159	0.0082	0.0058	-0.1165
478	Raemelton Farm	2013	Ginkgo biloba Saratoga	Gymnosperm	Asian	0	0	0	0.0023	0	-1
479	Raemelton Farm	2013	Gleditsia triacanthos Shademaster	Angiosperm	Non Asian	0.0119	0.5079	0.496	0.0163	0.033	0.3831
480	Raemelton Farm	2013	Halesia tetraptera	Angiosperm	Non Asian	0.0069	0.6458	0.0417	0.0093	0.0126	0.2001
481	Raemelton Farm	2013	Hamamelis x intermedia Arnold Promise	Angiosperm	Hybrid	0	0.0083	0.0167	0.0078	0.001	-0.7567
482	Raemelton Farm	2013	Hamamelis x intermedia Diane	Angiosperm	Hybrid	0	0	0.0417	0.0062	0.0029	-0.3164
483	Raemelton Farm	2013	Hamamelis x intermedia Jelena	Angiosperm	Hybrid	0	0	0	0.0012	0	-1
484	Raemelton Farm	2013	Heptacodium miconioides	Angiosperm	Asian	0	0	0.037	0.0035	0.001	-0.5294
485	Raemelton Farm	2013	Hibiscus syriacus Blue Bird	Angiosperm	Asian	0	0.4259	0.6111	0.0035	0.0078	0.4223
486	Raemelton Farm	2013	Hibiscus syriacus Diana	Angiosperm	Asian	0	0	0.1111	0.0023	0.0019	-0.0399
487	Raemelton Farm	2013	Hibiscus syriacus Red Heart	Angiosperm	Asian	0	0.463	0.3519	0.0035	0.0058	0.2974
488	Raemelton Farm	2013	Hibiscus syriacus Satin Blue	Angiosperm	Asian	0	0.3472	1.4861	0.0047	0.0087	0.3501
489	Raemelton Farm	2013	Ilex opaca Jersey Princess	Angiosperm	Non Asian	0	0.1667	0	0.0023	0.001	-0.3684
490	Raemelton Farm	2013	Juniperus chinensis Torulosa	Gymnosperm	Asian	0	0	0	0.0023	0	-1
491	Raemelton Farm	2013	Koelreuteria paniculata	Angiosperm	Asian	0.0299	0.9274	1.0769	0.0151	0.0262	0.3145
492	Raemelton Farm	2013	Larix kaempferi	Gymnosperm	Asian	0	0	0	0.0023	0	-1
493	Raemelton Farm	2013	Liquidambar styraciflua	Angiosperm	Non Asian	0.0556	0.1667	0.3889	0.0023	0.0039	0.2974
494	Raemelton Farm	2013	Liquidambar styraciflua Hapdell	Angiosperm	Non Asian	0	0	0.1667	0.0008	0.0019	0.4694
495	Raemelton Farm	2013	Liquidambar styraciflua Moraine	Angiosperm	Non Asian	0	0.1296	0.1481	0.0035	0.0068	0.3659
496	Raemelton Farm	2013	Liquidambar styraciflua Rotundiloba	Angiosperm	Non Asian	0.0556	0	0.0278	0.0023	0.0029	0.1614
497	Raemelton Farm	2013	Magnolia liliiflora x stellata Ann	Angiosperm	Asian	0	0	0.0278	0.0023	0.001	-0.3684
498	Raemelton Farm	2013	Magnolia liliiflora x stellata Merrill	Angiosperm	Asian	0	0.1667	1.0278	0.0023	0.0049	0.3954
499	Raemelton Farm	2013	Magnolia x loebneri Leonard Messel	Angiosperm	Asian	0	1.0833	0.0278	0.0023	0.0029	0.1614
500	Raemelton Farm	2013	Malus domestica Crimson Crisp	Angiosperm	unknown	0	0.1389	2.7222	0.0023	0.0058	0.4694
501	Raemelton Farm	2013	Malus domestica Freedom	Angiosperm	unknown	0	0.2778	0.5667	0.0058	0.0117	0.3781
502	Raemelton Farm	2013	Malus domestica Liberty	Angiosperm	unknown	0.0079	0.0714	0.5556	0.0082	0.0146	0.3285
503	Raemelton Farm	2013	Malus Donald Wyman	Angiosperm	unknown	0	0.6528	0.7222	0.0093	0.0194	0.3954
504	Raemelton Farm	2013	Malus halliana Adirondack	Angiosperm	Asian	0.0093	0.0833	0.1389	0.007	0.0126	0.3334

	Site	Year	Plant Taxon	Classification	Origin	Egg masses per 1 min survey	Nymphs per 1 min survey	Adults per 1 min survey	*p*	*r*	*E**
505	Raemelton Farm	2013	Malus Molten Lava	Angiosperm	unknown	0	0.537	0.0278	0.007	0.0049	-0.1304
506	Raemelton Farm	2013	Malus Pink Princess	Angiosperm	unknown	0	0	0.0185	0.007	0.001	-0.7333
507	Raemelton Farm	2013	Malus Prairifire	Angiosperm	unknown	0	0.1528	0.2454	0.014	0.0233	0.2974
508	Raemelton Farm	2013	Malus sargentii Select A	Angiosperm	Asian	0.0222	0.15	0.5278	0.0117	0.0233	0.3781
509	Raemelton Farm	2013	Malus x zumi Calocarpa	Angiosperm	Asian	0.0079	0.3651	0.1667	0.0082	0.0155	0.357
510	Raemelton Farm	2013	Metasequoia glyptostroboides	Gymnosperm	Asian	0	0.4028	0.0139	0.0047	0.0029	-0.1817
511	Raemelton Farm	2013	Nyssa sylvatica	Angiosperm	Non Asian	0	0.0556	0.2222	0.0047	0.0058	0.1614
512	Raemelton Farm	2013	Nyssa sylvatica Wildfire	Angiosperm	Non Asian	0	0.0833	0.0833	0.0023	0.0029	0.1614
513	Raemelton Farm	2013	Oxydendrum arboreum	Angiosperm	Non Asian	0	0.2639	0	0.0047	0.001	-0.625
514	Raemelton Farm	2013	Parrotia persica	Angiosperm	Non Asian	0	0.0833	0	0.0023	0.0019	-0.0399
515	Raemelton Farm	2013	Parrotia persica Ruby Vase	Angiosperm	Non Asian	0	0.0833	0	0.0023	0.001	-0.3684
516	Raemelton Farm	2013	Picea meyeri	Gymnosperm	Asian	0	0	0	0.0023	0	-1
517	Raemelton Farm	2013	Picea omorika	Gymnosperm	Non Asian	0	0	0.0069	0.0093	0.001	-0.7931
518	Raemelton Farm	2013	Picea omorika Pendula	Gymnosperm	Non Asian	0	0	0	0.0058	0	-1
519	Raemelton Farm	2013	Picea orientalis Atrovirens	Gymnosperm	Non Asian	0	0	0.0278	0.0023	0.001	-0.3684
520	Raemelton Farm	2013	Picea orientalis Aurea Compacta	Gymnosperm	Non Asian	0	0	0	0.0035	0	-1
521	Raemelton Farm	2013	Picea orientalis Gracillis	Gymnosperm	Non Asian	0	0	0	0.0012	0	-1
522	Raemelton Farm	2013	Picea pungens Blue Diamond	Gymnosperm	Non Asian	0	0.0139	0	0.0047	0.001	-0.625
523	Raemelton Farm	2013	Picea pungens Fastigiata	Gymnosperm	Non Asian	0	0	0	0.0023	0	-1
524	Raemelton Farm	2013	Picea pungens Fat Albert	Gymnosperm	Non Asian	0	0	0	0.0093	0	-1
525	Raemelton Farm	2013	Picea pungens Glauca Iseli Fastigata	Gymnosperm	Non Asian	0	0	0	0.0023	0	-1
526	Raemelton Farm	2013	Picea pungens Glauca Majestic Blue	Gymnosperm	Non Asian	0	0	0	0.0023	0	-1
527	Raemelton Farm	2013	Picea pungens Glauca Van Sikes	Gymnosperm	Non Asian	0	0	0	0.007	0	-1
528	Raemelton Farm	2013	Picea pungens Hoopsii	Gymnosperm	Non Asian	0	0	0	0.0047	0	-1
529	Raemelton Farm	2013	Pinus bungeana	Gymnosperm	Asian	0	0	0	0.0023	0	-1
530	Raemelton Farm	2013	Pinus cembra Chalet	Gymnosperm	Non Asian	0	0	0	0.0023	0	-1
531	Raemelton Farm	2013	Pinus cembra Silver Sheen	Gymnosperm	Non Asian	0	2.0556	0.1111	0.0012	0.0019	0.2974
532	Raemelton Farm	2013	Pinus densiflora Umbraculifera	Gymnosperm	Asian	0	0	0	0.0012	0	-1
533	Raemelton Farm	2013	Pinus flexilis Vanderwolf's Pyramid	Gymnosperm	Non Asian	0	0	0	0.007	0	-1
534	Raemelton Farm	2013	Pinus koraiensis	Gymnosperm	Asian	0	0	0	0.0047	0	-1
535	Raemelton Farm	2013	Pinus koraiensis Morris Blue	Gymnosperm	Asian	0	0.0139	0	0.0047	0.001	-0.625
536	Raemelton Farm	2013	Pinus nigra Arnold Sentinel	Gymnosperm	Non Asian	0	0	0	0.0012	0	-1
537	Raemelton Farm	2013	Pinus strobus Pendula	Gymnosperm	Non Asian	0	0	0	0.0023	0	-1
538	Raemelton Farm	2013	Pinus thunbergii Thunderhead	Gymnosperm	Asian	0	0	0	0.0012	0	-1
539	Raemelton Farm	2013	Platanus x acerifolia Bloodgood	Angiosperm	Non Asian	0.0231	1.4583	0.4213	0.014	0.0252	0.3334
540	Raemelton Farm	2013	Platanus x acerifolia Yarwood	Angiosperm	Non Asian	0.0139	1.25	0.1111	0.0047	0.0078	0.2974
541	Raemelton Farm	2013	Prunus avium BaDa Bing	Angiosperm	Non Asian	0	0.2222	0.1389	0.0023	0.0039	0.2974
542	Raemelton Farm	2013	Prunus avium Stella	Angiosperm	Non Asian	0	0	0.0556	0.0012	0.001	-0.0399
543	Raemelton Farm	2013	Prunus cerasifera Crimson Pointe	Angiosperm	Non Asian	0	0.0556	0.8889	0.0023	0.0039	0.2974
544	Raemelton Farm	2013	Prunus cerasifera Cripoizam	Angiosperm	Non Asian	0.0079	0.2302	0.0714	0.0082	0.0068	-0.0399
545	Raemelton Farm	2013	Prunus cerasifera Thundercloud	Angiosperm	Non Asian	0	0.0093	0.0093	0.007	0.0019	-0.5294
546	Raemelton Farm	2013	Prunus cerasus	Angiosperm	Non Asian	0	0.0556	0.1944	0.0023	0.0039	0.2974

	Site	Year	Plant Taxon	Classification	Origin	Egg masses per 1 min survey	Nymphs per 1 min survey	Adults per 1 min survey	*p*	*r*	*E**
547	Raemelton Farm	2013	Prunus cerasus Montmorency	Angiosperm	Non Asian	0	0.0556	0.2778	0.0012	0.0029	0.4694
548	Raemelton Farm	2013	Prunus persica Red Haven	Angiosperm	Asian	0	0.1778	1.5556	0.0058	0.0117	0.3781
549	Raemelton Farm	2013	Prunus sargentii	Angiosperm	Asian	0.0278	0.037	0.0741	0.007	0.0078	0.1035
550	Raemelton Farm	2013	Prunus serrula Tibetica	Angiosperm	Asian	0	0	0	0.0012	0	-1
551	Raemelton Farm	2013	Prunus serrulata Kwanzan	Angiosperm	Asian	0	0.3611	0.1667	0.0047	0.0087	0.3501
552	Raemelton Farm	2013	Prunus serrulata Snowgoose	Angiosperm	Asian	0.0069	0.1181	0.1667	0.0093	0.0097	0.0715
553	Raemelton Farm	2013	Prunus subhirtella Pendula	Angiosperm	Asian	0.0139	0.0833	0.5694	0.0047	0.0087	0.3501
554	Raemelton Farm	2013	Prunus subhirtella Pisnshzam	Angiosperm	Asian	0.0278	0.0694	0	0.0047	0.0049	0.0715
555	Raemelton Farm	2013	Prunus x incam Okame	Angiosperm	Asian	0.0079	0.1984	0.0159	0.0082	0.0058	-0.1165
556	Raemelton Farm	2013	Prunus x yedoensis	Angiosperm	Asian	0.0556	0.1667	0.3333	0.0012	0.0029	0.4694
557	Raemelton Farm	2013	Pseudocydonia sinensis	Angiosperm	Asian	0	0.0833	0.1389	0.0023	0.0029	0.1614
558	Raemelton Farm	2013	Pyrus betulifolia	Angiosperm	Asian	0.0278	0.1389	0.2222	0.0023	0.0058	0.4694
559	Raemelton Farm	2013	Pyrus communis Blake's Pride	Angiosperm	Non Asian	0	0	0.5556	0.0012	0.0029	0.4694
560	Raemelton Farm	2013	Pyrus communis Sunrise	Angiosperm	Non Asian	0	0	0.1667	0.0012	0.0019	0.2974
561	Raemelton Farm	2013	Pyrus fauriei Westwood	Angiosperm	Asian	0	0	0.1111	0.0012	0.001	-0.0399
562	Raemelton Farm	2013	Quercus acutissima	Angiosperm	Asian	0.0278	0.5833	0	0.0023	0.0019	-0.0399
563	Raemelton Farm	2013	Quercus alba	Angiosperm	Non Asian	0	0.3333	0.1111	0.0023	0.0019	-0.0399
564	Raemelton Farm	2013	Quercus coccinea	Angiosperm	Non Asian	0.0069	0.0556	0.1389	0.0093	0.0107	0.1187
565	Raemelton Farm	2013	Quercus robur Fastigiata	Angiosperm	Non Asian	0.1111	0.75	0	0.0023	0.0049	0.3954
566	Raemelton Farm	2013	Quercus robur Regal Prince	Angiosperm	Non Asian	0	0.1852	0.0247	0.0105	0.0049	-0.322
567	Raemelton Farm	2013	Quercus rubra	Angiosperm	Non Asian	0.0139	0.4028	0.066	0.0186	0.0233	0.1614
568	Raemelton Farm	2013	Rhus typhina Bailtiger	Angiosperm	Non Asian	0	0	0	0.0023	0	-1
569	Raemelton Farm	2013	Sambucus nigra Eva	Angiosperm	Non Asian	0	0	0	0.0023	0	-1
570	Raemelton Farm	2013	Sophora japonica Millstone	Angiosperm	Asian	0.0556	6.1389	1.7778	0.0023	0.0058	0.4694
571	Raemelton Farm	2013	Sophora japonica Regent	Angiosperm	Asian	0.0171	1.5299	0.5897	0.0151	0.034	0.4262
572	Raemelton Farm	2013	Stewartia koreana	Angiosperm	Asian	0	0	0.0833	0.0023	0.0019	-0.0399
573	Raemelton Farm	2013	Stewartia pseudocamellia	Angiosperm	Asian	0	0.0185	0.1296	0.007	0.0097	0.2122
574	Raemelton Farm	2013	Styrax japonicus	Angiosperm	Asian	0	0.137	0.0407	0.0175	0.0097	-0.238
575	Raemelton Farm	2013	Styrax obassia	Angiosperm	Asian	0	0	0.0278	0.0023	0.001	-0.3684
576	Raemelton Farm	2013	Syringa pekinensis Morton	Angiosperm	Asian	0.0159	0.1667	1.2302	0.0082	0.0175	0.4072
577	Raemelton Farm	2013	Syringa pekinensis Zhang Zhiming	Angiosperm	Asian	0	0.5556	4.6944	0.0023	0.0058	0.4694
578	Raemelton Farm	2013	Taxus x media Hicksii	Gymnosperm	Hybrid	0	0	0	0.0023	0	-1
579	Raemelton Farm	2013	Tetradium daniellii	Angiosperm	Asian	0.0278	1.1944	0.0833	0.0047	0.0107	0.4348
580	Raemelton Farm	2013	Thuja plicata Atrovirens	Gymnosperm	Non Asian	0	0	0	0.0012	0	-1
581	Raemelton Farm	2013	Thuja plicata Emerald Cone	Gymnosperm	Non Asian	0	0	0	0.0023	0	-1
582	Raemelton Farm	2013	Thuja plicata Zebrina	Gymnosperm	Non Asian	0	0	0	0.0023	0	-1
583	Raemelton Farm	2013	Thuja standishii x plicata Green Giant	Gymnosperm	Hybrid	0	0	0.0741	0.0035	0.0019	-0.238
584	Raemelton Farm	2013	Tilia cordata Greenspire	Angiosperm	Non Asian	0.0123	0.7593	0.1049	0.0105	0.0165	0.2711
585	Raemelton Farm	2013	Tilia tomentosa Sterling	Angiosperm	Non Asian	0.0159	1.8889	0.2302	0.0082	0.0165	0.3831
586	Raemelton Farm	2013	Tsuga canadensis Pendula	Gymnosperm	Non Asian	0	0	0	0.0012	0	-1
587	Raemelton Farm	2013	Ulmus americana Princeton	Angiosperm	Non Asian	0.0222	0.7222	0.1778	0.0117	0.0194	0.2974
588	Raemelton Farm	2013	Ulmus americana Valley Forge	Angiosperm	Non Asian	0.0556	0.7361	0.3056	0.0047	0.0107	0.4348

	Site	Year	Plant Taxon	Classification	Origin	Egg masses per 1 min survey	Nymphs per 1 min survey	Adults per 1 min survey	p	r	E*
589	Raemelton Farm	2013	Ulmus parvifolia Emer I	Angiosperm	Asian	0	0	0	0.0012	0	-1
590	Raemelton Farm	2013	Ulmus parvifolia Emer II	Angiosperm	Asian	0	0.3111	0	0.0058	0.001	-0.6883
591	Raemelton Farm	2013	Xanthoceras sorbifolia	Angiosperm	Asian	0	0.1019	0.0278	0.007	0.0058	-0.0399
592	Ruppert East	2013	Acer rubrum October Glory	Angiosperm	Non Asian	0.0069	2.3125	0	0.063	0.1066	0.2717
593	Ruppert East	2013	Acer saccharum Commemoration	Angiosperm	Non Asian	0.0278	0.7222	0	0.0157	0.0203	0.1418
594	Ruppert East	2013	Acer saccharum Green Mountain	Angiosperm	Non Asian	0.0093	1.0972	0.0602	0.0945	0.1066	0.0758
595	Ruppert East	2013	Acer saccharum Legacy	Angiosperm	Non Asian	0.025	0.6667	0.0083	0.0525	0.0609	0.0898
596	Ruppert East	2013	Amelanchier x grandiflora Autumn Brilliance	Angiosperm	Non Asian	0.0324	1.9491	0	0.0945	0.1624	0.279
597	Ruppert East	2013	Betula nigra Heritage	Angiosperm	Non Asian	0.0556	0.75	0	0.0157	0.0203	0.1418
598	Ruppert East	2013	Carpinus betulus Fastigiata	Angiosperm	Non Asian	0.0208	1.75	0	0.063	0.0609	-0.0011
599	Ruppert East	2013	Cercis canadensis	Angiosperm	Non Asian	0.0556	2.4444	0	0.0315	0.0508	0.249
600	Ruppert East	2013	Crataegus viridis Winter King	Angiosperm	Non Asian	0	0.5185	0	0.0236	0.0203	-0.0599
601	Ruppert East	2013	Gleditsia triacanthos Shademaster	Angiosperm	Non Asian	0.0238	0.754	0.0238	0.0551	0.0761	0.1754
602	Ruppert East	2013	Koelreuteria paniculata	Angiosperm	Asian	0	1.1111	0	0.0157	0.0254	0.249
603	Ruppert East	2013	Malus Adams	Angiosperm	unknown	0.0278	1.2222	0	0.0315	0.0254	-0.092
604	Ruppert East	2013	Malus Prairifire	Angiosperm	unknown	0	1.2361	0	0.0315	0.0558	0.2931
605	Ruppert East	2013	Malus Spring Snow	Angiosperm	unknown	0	0.8333	0	0.0157	0.0152	-0.0011
606	Ruppert East	2013	Malus x zumi Calocarpa	Angiosperm	Asian	0	1.0139	0	0.0315	0.0305	-0.0011
607	Ruppert East	2013	Picea abies	Gymnosperm	Non Asian	0	0	0	0.063	0	-1
608	Ruppert East	2013	Prunus serrulata Kwanzan	Angiosperm	Asian	0	1.6111	0	0.0079	0.0102	0.1418
609	Ruppert East	2013	Quercus acutissima	Angiosperm	Asian	0	0.5556	0	0.0236	0.0152	-0.2011
610	Ruppert East	2013	Styrax japonicus	Angiosperm	Asian	0	0	0	0.0079	0	-1
611	Ruppert East	2013	Thuja occidentalis Smaragd	Gymnosperm	Non Asian	0	0.0139	0	0.0315	0.0051	-0.7148
612	Ruppert East	2013	Thuja standishii x plicata Green Giant	Gymnosperm	Hybrid	0	0.197	0.0076	0.0577	0.0254	-0.3759
613	Ruppert East	2013	Tilia cordata Greenspire	Angiosperm	Non Asian	0	0.2917	0.0139	0.0315	0.0305	-0.0011
614	Ruppert East	2013	Ulmus parvifolia Dynasty	Angiosperm	Asian	0	0.0278	0	0.0157	0.0051	-0.5008
615	Ruppert East	2013	Ulmus parvifolia Emer II	Angiosperm	Asian	0.0278	1.5556	0	0.0315	0.0203	-0.2011
616	Ruppert East	2013	Zelkova serrata Village Green	Angiosperm	Asian	0.0046	0.1481	0	0.0945	0.0508	-0.2867
617	Ruppert North	2013	Acer rubrum Armstrong	Angiosperm	Non Asian	0.0347	1.5069	0.0278	0.036	0.0603	0.2699
618	Ruppert North	2013	Acer rubrum Franksred	Angiosperm	Non Asian	0.0278	1.2778	0.0139	0.1081	0.1055	0.0073
619	Ruppert North	2013	Acer rubrum October Glory	Angiosperm	Non Asian	0.0488	1.9634	0.0325	0.0616	0.0905	0.2086
620	Ruppert North	2013	Acer rubrum Sun Valley	Angiosperm	Non Asian	0	0.4444	0	0.009	0.0151	0.2699
621	Ruppert North	2013	Acer saccharum Commemoration	Angiosperm	Non Asian	0.0278	2.1667	0	0.009	0.0151	0.2699
622	Ruppert North	2013	Acer saccharum Green Mountain	Angiosperm	Non Asian	0.0556	0.9444	0.0069	0.036	0.0452	0.1321
623	Ruppert North	2013	Betula nigra BNMTF	Angiosperm	Non Asian	0	0.75	0.0139	0.018	0.0101	-0.266
624	Ruppert North	2013	Crataegus viridis Winter King	Angiosperm	Non Asian	0.0278	1.6019	0.0185	0.027	0.0377	0.1835
625	Ruppert North	2013	Cryptomeria japonica	Gymnosperm	Asian	0	0.0278	0.0278	0.009	0.0025	-0.5505
626	Ruppert North	2013	Cryptomeria japonica Yoshino	Gymnosperm	Asian	0	0	0	0.009	0	-1
627	Ruppert North	2013	Cupressocyparis leylandii	Gymnosperm	Non Asian	0	0.3542	0.2083	0.012	0.0151	0.1321
628	Ruppert North	2013	Gleditsia triacanthos Shademaster	Angiosperm	Non Asian	0.0056	0.4833	0.0611	0.045	0.0452	0.0213
629	Ruppert North	2013	Gleditsia triacanthos Skyline	Angiosperm	Non Asian	0.0139	0.5556	0.0972	0.036	0.0452	0.1321
630	Ruppert North	2013	Ilex x aquipernyi Meschick	Angiosperm	Hybrid	0	0.0333	0.0111	0.045	0.0176	-0.4226

	Site	Year	Plant Taxon	Classification	Origin	Egg masses per 1 min survey	Nymphs per 1 min survey	Adults per 1 min survey	*p*	*r*	*E**
631	Ruppert North	2013	Liquidambar styraciflua Cherokee	Angiosperm	Non Asian	0.0139	0.0972	0.0139	0.018	0.0101	-0.266
632	Ruppert North	2013	Malus Prairifire	Angiosperm	unknown	0.0556	1.8333	0	0.009	0.0126	0.1835
633	Ruppert North	2013	Ostrya virginiana	Angiosperm	Non Asian	0	0.0556	0.0278	0.009	0.005	-0.266
634	Ruppert North	2013	Picea abies	Gymnosperm	Non Asian	0	0	0	0.009	0	-1
635	Ruppert North	2013	Platanus x acerifolia Bloodgood	Angiosperm	Non Asian	0.0156	1.0111	0.0511	0.1126	0.1231	0.0638
636	Ruppert North	2013	Prunus cerasifera Thundercloud	Angiosperm	Non Asian	0.0278	0.1667	0.0278	0.009	0.005	-0.266
637	Ruppert North	2013	Prunus sargentii	Angiosperm	Asian	0.0278	0.3333	0.0833	0.009	0.0126	0.1835
638	Ruppert North	2013	Prunus serrulata Kwanzan	Angiosperm	Asian	0.0417	0.3472	0.2083	0.018	0.0276	0.2291
639	Ruppert North	2013	Prunus subhirtella Autumnalis	Angiosperm	Asian	0.0278	1.5278	0	0.009	0.0126	0.1835
640	Ruppert North	2013	Prunus subhirtella Pendula	Angiosperm	Asian	0	0.2222	0	0.009	0.0075	-0.0697
641	Ruppert North	2013	Prunus x incam Okame	Angiosperm	Asian	0.0139	0.6528	0.0556	0.018	0.0126	-0.1596
642	Ruppert North	2013	Prunus x yedoensis	Angiosperm	Asian	0.0486	0.9375	0.0625	0.036	0.0352	0.0073
643	Ruppert North	2013	Pyrus calleryana Cleveland Select	Angiosperm	Asian	0.0417	0.9861	0.0833	0.018	0.0226	0.1321
644	Ruppert North	2013	Quercus bicolor	Angiosperm	Non Asian	0	0.0833	0.0278	0.009	0.0075	-0.0697
645	Ruppert North	2013	Quercus palustrus Green Pillar	Angiosperm	Non Asian	0.0139	0.2639	0.0139	0.018	0.0075	-0.3939
646	Ruppert North	2013	Syringa reticulata Ivory Silk	Angiosperm	Asian	0.0278	0.75	0.0556	0.009	0.0126	0.1835
647	Ruppert North	2013	Thuja standishii x plicata Green Giant	Gymnosperm	Hybrid	0	0	0.2167	0.015	0.0176	0.0981
648	Ruppert North	2013	Tilia americana Redmond	Angiosperm	Non Asian	0	0.0139	0	0.018	0.0025	-0.7468
649	Ruppert North	2013	Tilia cordata Greenspire	Angiosperm	Non Asian	0.0222	0.2889	0.0056	0.045	0.0302	-0.1794
650	Ruppert North	2013	Tilia tomentosa Sterling	Angiosperm	Non Asian	0.1026	2.9872	0.0641	0.0195	0.0276	0.1909
651	Ruppert North	2013	Ulmus americana Princeton	Angiosperm	Non Asian	0	0.1667	0.0278	0.009	0.005	-0.266
652	Ruppert North	2013	Ulmus parvifolia Dynasty	Angiosperm	Asian	0	1.0556	0.0833	0.009	0.0075	-0.0697
653	Ruppert North	2013	Ulmus Patriot	Angiosperm	Hybrid	0.0278	2.6944	0.0278	0.009	0.0126	0.1835
654	Ruppert North	2013	Zelkova serrata Green Vase	Angiosperm	Asian	0.0123	0.0494	0	0.0405	0.0226	-0.266
655	Ruppert North	2013	Zelkova serrata Village Green	Angiosperm	Asian	0.0602	0.6343	0.0139	0.0541	0.0553	0.0305
